# Balancing Savanna Ungulate Diversity and Biomass: Optimal Human Use, Landscape Features, and Vegetation Types Under Varying Rainfall and Land Use

**DOI:** 10.1002/ece3.73501

**Published:** 2026-04-20

**Authors:** Gundula S. Bartzke, Joseph O. Ogutu, Hans‐Peter Piepho, Claire Bedelian, Michael E. Rainy, Russel L. Kruska, Jeffrey S. Worden, Kamau Kimani, Michael J. McCartney, Leah Ng'ang'a, Jeniffer Kinoti, Evanson C. Njuguna, Cathleen J. Wilson, Richard Lamprey, N. Thompson Hobbs, Robin S. Reid

**Affiliations:** ^1^ Biostatistics Unit, Institute of Crop Science, Faculty of Agricultural Sciences University of Hohenheim Stuttgart Baden‐Württemberg Germany; ^2^ International Livestock Research Institute Nairobi Nairobi County Kenya; ^3^ Mercy Corps Nairobi Nairobi County Kenya; ^4^ World Wildlife Fund Nairobi Nairobi County Kenya; ^5^ Campfire Conservation Nairobi Nairobi County Kenya; ^6^ Department of Infrastructure, Lands and Urban Development County Government of Laikipia Rumuruti Laikipia Kenya; ^7^ Department of Natural Resources, Faculty of Geo‐Information Science and Earth Observation University of Twente Enschede Overste the Netherlands; ^8^ Natural Resource Ecology Laboratory, Department of Ecosystem Science and Sustainability Colorado State University Fort Collins Colorado USA

**Keywords:** biomass, disturbance, diversity, environment, savanna, ungulates

## Abstract

To manage savanna ungulate communities in a changing environment, it is crucial to understand how their diversity and biomass change in relation to anthropogenic and environmental variables under different land uses and varying rainfall patterns. To this end, we analyzed fine‐scale data collected during the drought year of 1999 and the year with normal rainfall, 2002, in the Maasai Mara National Reserve and on adjacent pastoral lands in Kenya. Ungulate diversity peaked within 2–4 km of bomas, settlements, and 1–3 km of water sources. In the year with normal rainfall, ungulate species richness decreased in the presence of sheep and goats, though it increased with fire. In the drought year, ungulate biomass increased with grass color and shrub cover in the Mara Reserve and with tree cover on pastoral lands. Species richness often peaked at lower levels of grass color, shrub, or tree cover than biomass did. Our results suggest that ungulate diversity peaks in pastoral landscapes when sheep and goat densities are low, and wildlife can access areas more than 3–4 km from settlements. Maintaining diverse vegetation resources in core protected areas and pastoral lands is essential to maintaining high ungulate species richness and minimizing biomass loss during drought years. This can be achieved by providing a variety of food sources and cover. Potential candidate sites for ungulate conservancies in the Mara region include landscapes with low slopes, elevations of up to 1700 m above sea level (masl), and locations within 2–3 km of water sources. Our results highlight how policy and governance can manage these variables to maximize ungulate diversity and minimize biomass loss in contexts where disturbance and resource supply are coupled. This can help alleviate the tension between pastoral use and conservation in protected areas and human‐dominated African savannas.

## Graphical abstract

This study analyzes the relationship between anthropogenic and environmental variables and ungulate diversity and biomass in Kenya's Mara Ecosystem. The results suggest that ungulate species richness peaks in pastoral landscapes with access to areas more than 3–4 km from settlements but decreases in the presence of sheep and goats. Maintaining high ungulate diversity and minimizing the impact of droughts on ungulate biomass in savannas requires effective policies and governance to preserve diverse vegetation and ensure access to water in protected areas and pastoral lands.

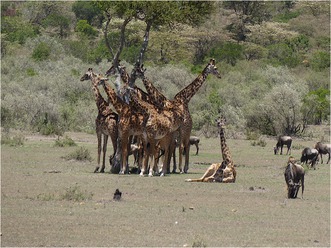

## 1 Introduction

Ungulate communities underpin key ecosystem functions in African savannas. They mediate vegetation structure, seed dispersal, nutrient cycling, and energy flow (McNaughton 1984; McNaughton et al. 1988; Detling 1988; Dublin et al. 1990; Seagle et al. 1992; Sinclair 1995; Miller 1996; Du Toit and Cumming 1999). These communities also sustain carnivore and scavenger guilds (Sinclair et al. 2003). These functions may depend on how total abundance and species diversity change with gradients of human use and resource availability. Although ungulate abundance and diversity often decline with increasing settlements, livestock grazing, and infrastructure (Green et al. 2019; Kinnaird and O’brien 2012; Veldhuis et al. 2019), diversity can peak under intermediate human use (Bartzke et al. 2025) such as controlled livestock grazing (Kinnaird and O’brien 2012) or fire (Klop and van Goethem 2008; Klop and Prins 2008).

Intermediate disturbance can increase habitat heterogeneity without eliminating resources (Grime and Pierce 2012). Resource supply can also vary independently of human use. For example, rainfall‐mediated water availability regulates primary productivity. Low rainfall limits the quantity (Boutton et al. 1988), while high rainfall limits the quality (Georgiadis and McNaughton 1990) of food for ungulates. This coupled disturbance–resource supply setting raises central questions for adaptive conservation: How are ungulate diversity and biomass related to human use and environmental variables under varying climates and land use? How can these levers be managed to maximize ungulate diversity and minimize the loss of ungulate biomass?

In our previous work (Bartzke et al. 2025), we used the intermediate disturbance hypothesis (Connell 1978) and the humped‐back model of species richness and biomass production (Grime and Pierce 2012) to frame expected diversity–biomass trade‐offs along disturbance and resource gradients. Building on that foundation, we expanded our analyses from spatial patterns relative to a protected area boundary to a set of 26 landscape, vegetation, water, climate, and human‐use variables, some of which are intercorrelated (Bartzke et al. 2025). Although the diversity, abundance, and occurrence of ungulates have been linked to distinct variables (Treydte et al. 2009, 2013; Ogutu et al. 2010; Green et al. 2015, 2019; Xu and Butt 2024; Bierhoff et al. 2024), we identified the most consistent variables associated with the largest change in ungulate diversity and biomass. One of our goals was to determine whether distance to pastoralist settlements (bomas) is a central predictor. Bomas integrate multiple co‐occurring disturbances, such as livestock grazing, human presence, habitat modification, and associated infrastructure, whose intensity typically decays with distance (Ogutu et al. 2010).

Diversity should peak at levels of environmental variables associated with intermediate human use and resources where disturbance‐created heterogeneity is high (Grime and Pierce 2012) but competitive displacement should remain limited (Connell 1978). Ungulate biomass should peak at lower levels of human use where disturbance is lower and at higher levels of resources, where competitive displacement is higher than at the diversity optimum (Connell 1978; Grime and Pierce 2012). After a drought, we expect both diversity and biomass to shift toward lower levels of human use and higher levels of resources than under normal rainfall conditions. This is consistent with the idea that peak diversity shifts toward lower disturbance levels with decreasing productivity (Kondoh 2001) and with the idea that animals aggregate in areas with more resources as they become scarcer (Anderson et al. 2012).

Within this framework, we expect ungulate diversity to peak in environments with intermediate levels of resources related to rainfall, elevation, distance to water sources, cover, height, and vegetation color in savannas. Intermediate productivity environments provide more spatial and temporal niches (Grime and Pierce 2012). Peak diversity may also occur at intermediate distances from bomas because disturbances, such as livestock grazing, can create opportunities for subordinate species by reducing the abundance of dominant species (Connell 1978). Conversely, we expect ungulate biomass to peak farther from bomas and closer to water sources with higher levels of rainfall, vegetation cover, height, and color. This expectation is supported by the sensitivity of dominant grazers, such as wildebeest ( *Connochaetes taurinus* ) and zebra ( *Equus quagga* ), to land use (Kinnaird and O’brien 2012) and drought (Abraham et al. 2019). Additionally, ungulate biomass should decline more than diversity in the presence of livestock and fire because these factors truncate forage availability.

Our approach goes beyond merely demonstrating that diversity and biomass vary across landscapes (Bartzke et al. 2025) to identifying the variables that most constrain these community attributes and the most effective ways to mitigate the impacts of drought. This enables us to recommend the most beneficial management actions. This variable‐centered inference can inform ungulate conservation strategies based on the expected relationships between disturbance, resources, and diversity or biomass (Bartzke et al. 2025). Our framework is grounded in the intermediate disturbance hypothesis (Connell 1978) and the humped‐back model of species richness and biomass production (Grime and Pierce 2012). While our results do not always align with these general expectations, departures from the predicted “humps” provide valuable information. These results can guide the management of human use and resource availability by establishing protected areas, controlling livestock grazing, and adapting infrastructure and fire management in a changing environment.

## 2 Methods

### 2.1 Data Collection

#### 2.1.1 Study Area

To predict changes in ungulate diversity and biomass in relation to human use, resource variables, and rainfall conditions (the drought year of 1999 vs. the year with normal rainfall, 2002), our study area encompassed large spatial gradients of human use, vegetation, rainfall, and elevation. The region included the Maasai Mara National Reserve and adjacent pastoral lands in southwestern Kenya (Bartzke et al. 2025: figure 2). The Mara Reserve and the adjacent Serengeti National Park to the south support an exceptional abundance and diversity of ungulates and other mammals (Mduma and Hopcraft 2008). For more details on the overlapping study areas in both census years, see Reid et al. (2003) and Ogutu et al. (2010, 2014).

#### 2.1.2 Field Data Collection and Preparation

During extensive and short fieldwork sessions in November, coordinated by the International Livestock Research Institute (ILRI), 1667 1‐km‐by‐1‐km blocks were surveyed. These included 13,695 333‐m‐by‐333‐m sub‐blocks in 1999 and 13,112 sub‐blocks in 2002 (Reid et al. 2003; Ogutu et al. 2010, 2014). Team members counted wild ungulates, livestock, carnivores, active and abandoned bomas, human infrastructure, fires, water sources, vehicles, and litter. They also recorded vegetation characteristics (Reid et al. 2003; Ogutu et al. 2010, 2014). For unsampled sub‐blocks, we used kriging (Bivand et al. 2008) to predict the cover, height, and color of grasses, shrubs, or trees. We estimated the total expected ungulate numbers for each block and species combination (Bartzke et al. 2025). We derived ungulate biomass from unit weights for each species (Coe et al. 1976), and derived five measures of ungulate diversity, ranging from raw species richness to species evenness (Hill 1973; Marcon and Hérault 2015).

We obtained rainfall data for each sub‐block during the wet and dry seasons, as well as for the preceding month, from the CHIRPS global rainfall dataset (Funk et al. 2015). Using QGIS (QGIS Development Team 2021) and ArcView (ESRI 2000), we extracted the following variables: slope, elevation, distance to the reserve boundary, bomas, water sources, and infrastructure. We averaged the following variables across all sub‐blocks per block: vegetation measures, rainfall, slope, elevation, and distance measures. The presence of livestock, carnivores, vehicles, or litter was noted for each block.

### 2.2 Data Analysis

We used boosted regression models with the gamboostLSS R package version 2.0.6 (Mayr et al. 2012; Hofner et al. 2016, 2022; Thomas et al. 2018) in R version 4.2.2 (R Core Team 2022). Based on empirical data of ungulate diversity or biomass, we applied appropriate statistical distributions and link functions, while accounting for spatial effects in each census year (Bartzke et al. 2025). Since each variable may be linked to a number of distribution parameters (Mayr et al. 2012) the machine learning model cannot easily indicate a variable's relative importance. Using stability selection (Meinshausen and Bühlmann 2010; Hofner et al. 2015) we identified consistent variables from a set of 26 possibly correlated predictor variables, depending on land use and census year (Bartzke et al. 2025). These variables may or may not be associated with a large change in ungulate diversity and biomass.

Cross‐validation via averaging model likelihoods was used to tune the models (Mayr et al. 2017), as measures such as root mean square error, *R* ^2^ and confidence intervals are still unavailable for complex boosting models with correlated predictors (Mayr and Hofner 2018). Error propagation may have occurred when random explanatory variables were treated as fixed, resulting in biased regression parameters (Zuur et al. 2009b). Methods that address this issue are computationally demanding (Faraway 2005; Zuur et al. 2009a) and have yet to be implemented for boosting models. Since the cross‐validated correlation coefficients between the observed and kriged values were greater than 0.5 for all but one variable, errors resulting from kriging vegetation variables were probably not substantial. Although the biomass estimates were potentially biased due to missed observations of individual ungulates in dense vegetation areas, the zero‐adjusted boosting models accounted for varying response errors with each predictor variable (Mayr et al. 2012).

From these cross‐validated models, we predicted how ungulate diversity and biomass varied with each variable (https://doi.org/10.5281/zenodo.17073514: R codes ‘mc_1km_pred.r’,mc_1km.RData, and ‘mc_1km_plots.r’). To predict ungulate diversity and biomass for a continuous variable's range, we set all other continuous variables to their median values due to their skewed distributions. Categorical variables were set to zero. To obtain category‐specific predictions, we set all other categorical variables to zero while holding the remaining variables constant at their medians. We only interpret predictions for the most consistent variables associated with the greatest change in ungulate diversity and biomass.

## 3 Results

The diversity and biomass of ungulates in the Mara fluctuate along a gradient of coupled disturbance and resources. Along this gradient, human use increases disturbance while water, forage, and landscape features determine resource availability. According to our framework (Bartzke et al. 2025), the intermediate disturbance hypothesis (Connell 1978) and the humped‐back model of species richness and biomass production (Grime and Pierce 2012) predict that diversity peaks where disturbance creates heterogeneity without overwhelming resources, whereas biomass peaks where resources are high and disturbance is low. These results focus on the subset of predictors that consistently explain the most variation in diversity and biomass.

### 3.1 Species Richness and Biomass of Savanna Ungulates in Relation to Human Uses

#### 3.1.1 Occupied Bomas

**FIGURE 1:**
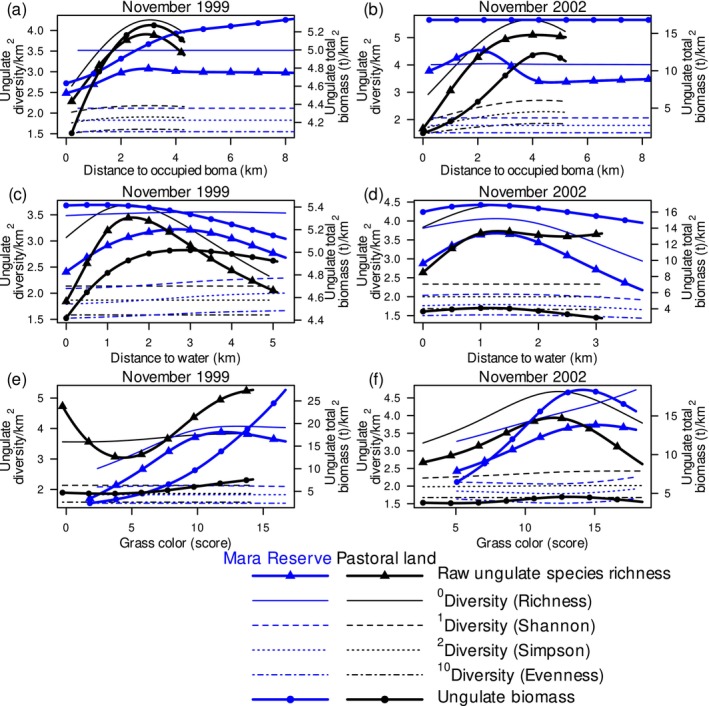
Ungulate diversity expressed as the raw species richness (continuous thick lines with triangles), diversity of orders 0 (richness: Continuous lines), 1 (Shannon: Dashed lines), 2 (Simpson: Dotted lines) and 10 (evenness: Dashed‐dotted lines), and biomass (continuous thick lines with dots) of savanna ungulates in relation to distance to the nearest occupied boma (a, b), water (c, d) and grass color (e, f) in the Maasai Mara National Reserve (blue lines) and adjacent pastoral lands (black lines) in Kenya in November of the 1999 drought year (a, c, e) and November of the 2002 normal rainfall year (b, d, f). Predictions are truncated at 8 km from the nearest occupied boma.

Distance to occupied bomas captures an integrated disturbance signal—livestock grazing, human presence, and localized habitat modification. Ungulate diversity peaked on pastoral lands between 3.8 and 4.4 km from occupied bomas in 2002 and at about 3 km from occupied bomas in 1999 (Figure 1a,b; Appendix S2: Figures S1a,b and S2a,b,e,f,i,j,m,n). This is consistent with an intermediate‐disturbance optimum. Raw species richness on pastoral lands exceeded that of the Mara Reserve beyond 2.3 km from bomas in 2002 and beyond 0.6 km from bomas in 1999 (Figure 1a,b; Appendix S1: Figure S1a,b). Bias‐adjusted diversity measures on pastoral lands exceeded those in the Mara Reserve beyond 0.4 to 1.3 km from bomas in 2002 and about 1 km from bomas in 1999 (Figure 1a,b; Appendix S1: Figure S2a,b,e,f,i,j,m,n). These results suggest that moderate human use can increase ungulate diversity on pastoral lands relative to reserves. Ungulate biomass on pastoral lands decreased by 87% within its peak at 4.4 km from occupied bomas in 2002. This indicates that disturbance suppresses dominant contributors to herbivore biomass. But biomass decreased only by 23% within its peak at 3.1 km from occupied bomas in 1999 (Figure 1a,b; Appendix S1: Figure S1c,d). Drought may compress habitat choice and draw ungulates toward settlement‐associated resource patches.

#### 3.1.2 Abandoned Bomas

**FIGURE 2:**
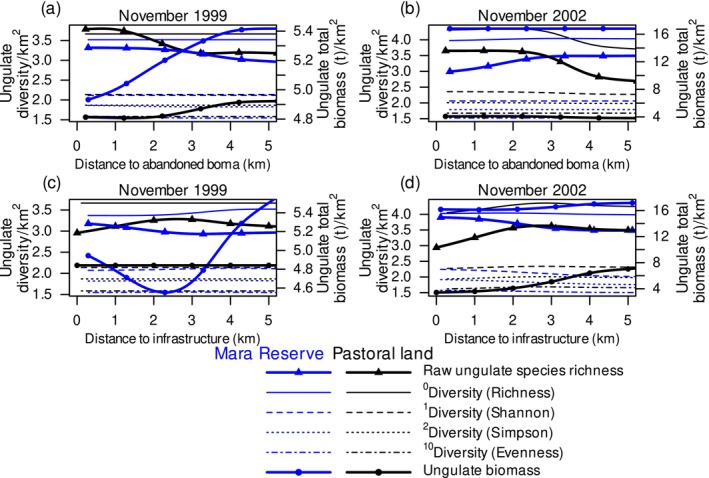
Ungulate diversity, expressed as the raw species richness (continuous thick lines with triangles), diversity of orders 0 (richness: Continuous lines), 1 (Shannon: Dashed lines), 2 (Simpson: Dotted lines) and 10 (evenness: Dashed‐dotted lines), or ungulate biomass (continuous thick lines with dots in) of savanna ungulates in relation to distance to the nearest abandoned boma (a, b) and infrastructure (c, d) in the Maasai Mara National Reserve (blue lines) and on the adjacent pastoral lands (black lines) in Kenya in November of the 1999 drought year (a, c) and November of the 2002 normal rainfall year (b, d). Predictions are truncated at 5 km from the nearest abandoned bomas or infrastructure.

The land around abandoned bomas may provide nutrients that increase local plant production and favor more diverse and abundant ungulate assemblages. Within 5 km of abandoned bomas, raw ungulate species richness increased by 34% and bias‐adjusted species richness increased by 17% on pastoral lands in 2002 (Figure 2b; Appendix S1: Figures S3b and S4b). In the Mara Reserve in 1999, raw species richness also increased by 12%, whereas ungulate biomass decreased by 9% within 5 km of abandoned bomas (Figure 2a; Appendix S1: Figure S3a,c). These changes suggest that nutrient enrichment may increase the number of species present without necessarily increasing total biomass.

#### 3.1.3 Infrastructure

Infrastructure represents an anthropogenic disturbance that can alter movement, cause avoidance, and block access to resources. Raw ungulate species richness decreased by 19% within 2.7 km of infrastructure, and ungulate biomass decreased by 51% within 5 km of infrastructure on pastoral lands in 2002 (Figure 2d; Appendix S1: Figure S3f,h). These results suggest that infrastructure suppresses both community richness and biomass. In the Mara Reserve, bias‐adjusted diversity measures, which account for species evenness, increased by between 5% and 11% within 5 km of infrastructure in 2002. These measures decreased by between 4% and 10% in 1999 (Figure 2c,d; Appendix S1: Figure S4g,h,k,l,o,p). Ungulate biomass decreased by 17% only at a distance of 2.3 km from infrastructure compared to 5.0 km in the Mara Reserve in 1999 (Figure 2c; Appendix S1: Figure S3g). These results suggest that the effects of infrastructure are context‐dependent and may shift with localized resource availability.

#### 3.1.4 Livestock

Changes in ungulate diversity and biomass varied according to livestock species composition, with sheep and goats showing a more consistent signal of competitive displacement than cattle. In the Mara Reserve, raw ungulate species richness decreased by 29% in 2002 where sheep and goats were observed (Figure 4b; Appendix S1: Figure S5b). On pastoral lands, both raw and bias‐adjusted ungulate species richness decreased by about 15% where sheep and goats were observed in 2002 (Figure 4b; Appendix S1: Figures S5b, S6b, and S7b). Cattle accounted for only 7%–16% of the total livestock biomass (Appendix S1: Figure S10a,b) and did not appear to contribute substantially to variations in ungulate diversity or biomass.

#### 3.1.5 Fire

Fire can cause intermediate disturbance, which resets the vegetation, increases nutrient concentration of forage and expands short‐grass patches. With fire, raw ungulate species richness increased by between 9% and 16% depending on land use. Bias‐adjusted species richness increased by 5% on pastoral lands in 2002 (Figure 4a,b; Appendix S1: Figures S5a,b and S7a). These changes are consistent with post‐burn regrowth increasing the presence of species that require concentrated nutrients without uniformly increasing biomass across functional groups.

#### 3.1.6 Vehicles and Litter

Vehicles and litter were mostly unstable predictors of ungulate diversity and biomass (Figure 4a,b; Appendix S1: Figures S6–S9a–d). These indicators may vary too locally or covary with stronger predictors, such as bomas and infrastructure.

### 3.2 Species Richness and Biomass of Savanna Ungulates in Relation to Environmental Resources

#### 3.2.1 Grass

Grass color approximates nutrient content and therefore aligns with the resource axis of the humped‐back framework. Ungulate biomass peaked in predominantly green areas (14 units of grass color score) in the Mara Reserve in 2002 (Figure 1f; Appendix S1: Figure S1d). In the drought year of 1999, ungulate biomass increased 12‐fold with increasing green grass from a score of 2 to 17 (Figure 1e; Appendix S1: Figure S1c). This indicates that grazing biomass concentrated into the remaining green forage patches. Ungulate species richness peaked in predominantly green areas or increased as grass became greener in the Mara Reserve in both census years and on pastoral lands in 2002 (Figure 1e,f; Appendix S1: Figures S1i,j and S11a,b; Appendix S2: Section S1.1). Diversity measures accounting for species evenness tended to increase as grass became greener in the Mara Reserve in 2002 (Figure 1f; Appendix S1: Figure S11f,j,n). These patterns suggest that increased greenness increases both species numbers and biomass, especially when drought limits green forage.

**FIGURE 3:**
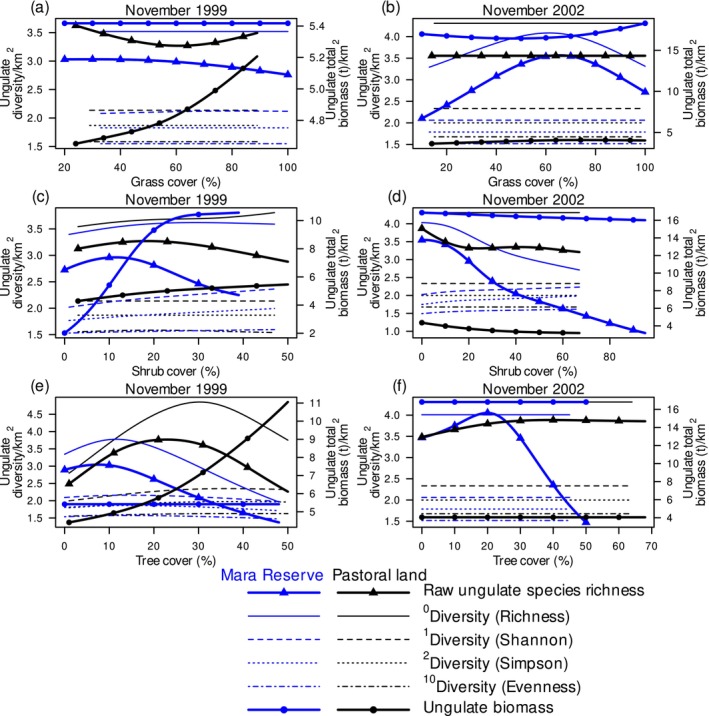
Ungulate diversity expressed as the raw species richness (continuous thick lines with triangles), diversity of orders 0 (richness: Continuous lines), 1 (Shannon: Dashed lines), 2 (Simpson: Dotted lines) and 10 (evenness: Dashed‐dotted lines), and biomass (continuous thick lines with dots) of savanna ungulates in relation to grass cover (a, b), shrub cover (c, d) and tree cover (e, f) in the Maasai Mara National Reserve (blue lines) and adjacent pastoral lands (black lines) in Kenya in November of the 1999 drought year (a, c, e) and November of the 2002 normal rainfall year (b, d, f).

Grass structure may further modulate ungulate diversity and biomass. Raw and bias‐adjusted ungulate species richness peaked at medium to high grass cover (between 61% and 65%) in the Mara Reserve in 2002 (Figure 3b; Appendix S1: Figures S11d and S12b). This is consistent with the idea that intermediate resource levels support more niches. Raw ungulate species richness, however, decreased by 55% as grass height increased to 0.7 m on pastoral lands in 2002 (Appendix S1: Figures S13b and S14b). This suggests that tall grass provides fewer nutrients for most species, shifting communities away from the diversity but not the biomass optimum.

#### 3.2.2 Water

Proximity to water may concentrate resources and risks. Most ungulate diversity measures in both census years and ungulate biomass in the normal rainfall year peaked between about 1 and 3 km from water in the Mara Reserve (Figure 1c,d; Appendix S1: Figures S1e,f,h and S2d,h,l,p). This indicates that intermediate distances balance access to drinking water while avoiding heavily grazed or disturbed riparian margins. Ungulate diversity that accounted for species evenness decreased by about 10% near water compared to 5.3 km from water in the reserve in the drought year of 1999 (Figure 1c, Appendix S1: Figure S2g,k,o). This is consistent with drought‐driven crowding that increases dominance of a subset of species. Ungulate biomass decreased by 12% within 2.8 km of water on pastoral lands in 1999 (Figure 1c; Appendix S1: Figure S1g). This suggests that forage depletion and disturbance outweigh the benefits of water.

#### 3.2.3 Slope and Elevation

Topography can influence microclimate, soil moisture and accessibility. Ungulate species richness and biomass mostly peaked at low slopes and low to medium elevations (Appendix S1: Figures S15a–d, S16a–h, and S17a–p; Appendix S2: Section S1.2). These patterns imply that flatter landscapes at lower elevations reduce the energetic costs of movement and provide more accessible forage, which supports ungulate diversity and biomass.

#### 3.2.4 Shrubs and Trees

Although woody vegetation can mitigate the effects of drought, it can also increase the risk of predation. Its effects may therefore be context dependent. Ungulate biomass increased in 1999 with rising shrub cover in the Mara Reserve and increasing tree cover on pastoral lands (Figure 3c,e; Appendix S1: Figure S12g,k; Appendix S2: Section S1.3). This is consistent with woody plants protecting green forage and providing browse and shade during drought. Ungulate diversity accounting for species evenness tended to increase with increasing shrub cover in the Mara Reserve in both census years (Figure 3d,e; Appendix S1: Figure S18e,f,i,j,m,n; Appendix S2: Section S1.3). In contrast, both raw and bias‐adjusted species richness decreased with increasing shrub cover in the Mara Reserve in 2002 (Figure 3d; Appendix S1: Figures S12f and S18b; Appendix S2: Section S1.3). This indicates that woody vegetation can reduce the number of savanna specialists under normal rainfall conditions. Ungulate diversity tended to peak at low to moderate (between 8% and 40%) tree cover depending on land use and census year (Figure 3e,f; Appendix S1: Figures S12i,j and S18c,g,j,k,o). This is consistent with a humped response along a resource axis.

Ungulate diversity and biomass also change with structural woody attributes. Shannon and Simpson diversity in the Mara Reserve in 2002, as well as ungulate diversity measures accounting for species evenness on pastoral lands in 1999, peaked at about 1.5 m shrub height (Appendix S1: Figures S13c,d, S14e,f and S19e,f,i,j,m). This indicates that low woody layers increase heterogeneity without creating dense cover. Ungulate diversity decreased with increasing tree height on pastoral lands in 2002 (Appendix S1: Figures S13f, S14j and S20b,f,j,n; Appendix S2: Section S1.3). In the Mara Reserve, ungulate biomass had a low point at 6.4 m tree height in the in 2002 (Appendix S1: Figures S13f and S14l; Appendix S2: Section S1.3) and the bias‐adjusted species richness peaked at 2.8 m tree height in 1999 (Appendix S1: Figures S13e and S20a). This indicates that intermediate woody stature can support diversity, whereas certain structural states reduce biomass by limiting grass production.

The color of woody vegetation provides a complementary signal of available resources. On pastoral lands, bias‐adjusted ungulate diversity increased with greener shrubs in 2002, while species richness and Shannon diversity increased with greener trees in 1999 (Appendix S1: Figures S19d,h,l,p, S20c,g, S21b,c, and S22i; Appendix S2: Section S1.3). In the Mara Reserve, species richness peaked at medium greenness of trees in 2002, and ungulate diversity, except for evenness, at medium to high greenness of trees in 1999 (Appendix S1: Figures S20c,d,g,k,o, S21c,d, and S22i,j; Appendix S2: Section S1.3). These results are consistent with green woody patches functioning as drought refugia or nutrient hotspots.

#### 3.2.5 Rainfall

Raw species richness increased by 67% and bias‐adjusted species richness by 28% as wet season rainfall in the Mara Reserve increased from about 500 to 744 mm in 1999 (Appendix S1: Figures S23a, S24a, and S25a). Raw species richness peaked at intermediate to high wet season rainfall on pastoral lands in 1999 (Appendix S1: Figures S23a and S24a). Ungulate biomass peaked at intermediate dry season rainfall in the Mara Reserve in 2002. Ungulate species richness peaked at intermediate preceding month's rainfall on pastoral lands in 2002 (Appendix S1: Figures S23d,f, S24h,j, and S26b). These peaks are consistent with a humped response to resources when rainfall increases beyond the point at which the nutrient content of forage falls behind its quantity.

#### 3.2.6 Carnivores

**FIGURE 4:**
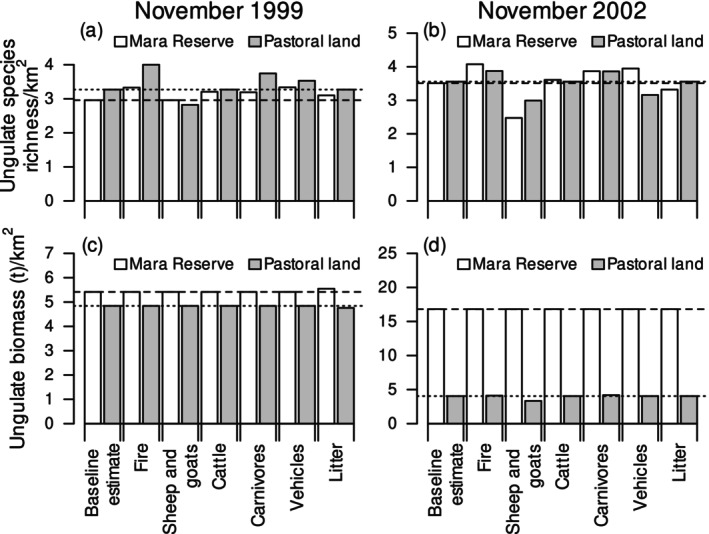
Raw species richness (a, b) and biomass (c, d) of savanna ungulates in relation to the presence of fire, livestock, carnivores, vehicles and litter in the Maasai Mara National Reserve (white bars) and on the adjacent pastoral lands (gray bars) in November of the drought year of 1999 (a, c) and November of the normal rainfall year of 2002 (b, d).

Carnivores were mostly unstable predictors of ungulate diversity and biomass (Figure 4a,b; Appendix S1: Figures S6a,b and S8a). This suggests that predation risk is inconsistent at the analyzed scale, varies with stronger habitat predictors, or influences behaviors and movements that census‐based co‐occurrence does not fully capture.

### 3.3 Summary of Important Variables for Ungulate Diversity and Biomass

The most important variables for ungulate diversity and biomass across models were occupied bomas, distance to water sources, grass color, slope, and elevation. These predictors align with the disturbance–resource framework: bomas and infrastructure concentrate disturbance, while proximity to water, grass color, and topography structure access to resources. Other important human use variables were abandoned bomas, infrastructure, and the presence of sheep and goats as well as fire. These predictors also modify disturbance intensity or resource availability. Grass, shrub, and tree cover and height were intermittently important, indicating context dependence and possible trade‐offs between open‐savanna specialists and drought buffering. Shrub and tree color or rainfall were less consistently related to ungulate diversity and biomass. These findings support the interpretation that climatic effects are mediated by habitat structure and human use. While the direction and magnitude of change varied according to land use and the year of the census, the results generally align with the ecological pattern. Diversity peaks at intermediate disturbance and intermediate‐to‐high resources. In contrast, biomass is most sensitive to resource constraints and therefore shifts more strongly under drought.

## 4 Discussion

The structure of the ungulate community in the Mara varies with a coupled disturbance–resource supply system. While several relationships between ungulate diversity, biomass and human use or resources diverged from our a priori expectations, dominant signals suggest that biomass tracks resource availability more closely than diversity does. Diversity peaks under intermediate disturbance and intermediate‐to‐high resource availability. Through the intermediate‐disturbance and humped‐back frameworks, these patterns indicate that disturbance and resource limitations weaken competitive asymmetries and create niches. In the drought year, peaks in both diversity and biomass shifted toward higher resource availability and lower human use. This indicates that drought compresses suitable space and amplifies the costs of disturbance. One implication for management is that a subset of variables consistently predicts diversity and biomass, while many other predictors either do not contribute, contribute weakly, or contribute only in certain contexts. The resulting synthesis provides a basis for prioritizing interventions by targeting important, interpretable predictors and treating less consistent predictors as modifiers, either alone or in combination with other pressures.

### 4.1 Ungulate Diversity and Biomass Decline Close to Occupied Bomas, Whereas Ungulate Species Richness Can Increase Close to Abandoned Bomas and With Fire

Occupied bomas emerge as one of the most consistent predictors of decreasing ungulate diversity and biomass on pastoral lands. This suggests that disturbance centered on settlements should be the primary focus of conservation strategies. Higher ungulate diversity on pastoral lands compared to the Mara Reserve beyond about 1 and 2 km from bomas supports the intermediate‐disturbance hypothesis. This hypothesis coincides with peak diversity at intermediate levels of human use. The increase in ungulate biomass away from bomas in the drought year, as well as the lower biomass on pastoral lands compared to the reserve under normal rainfall conditions, supports the expectation that biomass peaks where human use is lower than the diversity optimum. These patterns imply that diversity can be sustained under moderate use but biomass erodes as disturbance intensifies. Our results suggest maintaining a spatial envelope in which disturbance creates heterogeneity without overwhelming the supply of resources. The optimal distance may lie between 3 and 4.4 km from bomas to maximize ungulate diversity while minimizing biomass loss.

Reducing the number of livestock in bomas would likely benefit ungulates. Grazing pressure near occupied bomas can reduce forage, which may have contributed to the decline of ungulate species and total biomass. Our results suggest that managing sheep and goats would be the most effective approach. While some small‐ to medium‐sized species may benefit from grazed habitats (Bhola et al. 2012), sheep and goats appear to displace other ungulates. Cattle represented only a small proportion of livestock biomass and do not appear to reduce ungulate diversity or biomass (Xu and Butt 2024). This differentiation matters for governance: it points to targeted, socially feasible adjustments—stocking composition and herd management—rather than blanket restrictions that undermine livelihoods and erode compliance.

Conservation strategies should anticipate the convergence of wild and domestic ungulates in response to drought. The less severe decline in ungulate species richness and biomass near occupied bomas in the drought year compared to the year with normal rainfall contradicts the expectation that both metrics would peak at lower disturbance under drought. This departure is informative because it suggests that resource scarcity and limited access to refugia may draw ungulates toward bomas. Pastoralists may track the same resource gradients that benefit their livestock (Western and Dunne 1979), and ungulates may form larger groups near bomas under dry conditions (Leweri et al. 2022). In the intermediate‐disturbance and humped‐back frameworks, drought increases the cost of being far from resources. This could temporarily override the avoidance of people and convert bomas from disturbance sources into markers of residual resources. Management should mitigate conflicts and control access to grazing areas accordingly.

Abandoned bomas can provide nutrient‐rich sites (Muchiru et al. 2009) with increased plant species richness, grass or forb biomass, and shrub cover in the surrounding areas (Muchiru et al. 2008, 2009). With adequate moisture, these sites can create diverse niches and increase ungulate species richness. This was observed within 5 km of abandoned bomas on pastoral lands in 2002 and in the Mara Reserve in 1999. However, the benefits of nutrient enrichment diminished under water limitations. Ungulate species richness increased less near abandoned bomas in the drought year, implying that nutrient enrichment alone cannot offset drought‐driven constraints.

Similarly, infrastructure may interact with resources. The net effect may depend on whether the infrastructure fragments access to resources or coincides with resource‐rich habitats. For example, increased rainfall or greener vegetation near infrastructure on pastoral lands in the drought year could have offset the decrease in ungulate biomass near infrastructure compared to the year with normal rainfall. The change in bias‐adjusted diversity measures, which account for species evenness, was inconsistent near infrastructure between 1999 and 2002. But the decrease in ungulate species richness and biomass near infrastructure on pastoral lands in 2002 suggests that reducing infrastructure could benefit ungulate diversity and biomass (Green et al. 2019).

Finally, our results support the use of fire during normal rainfall to promote ungulate diversity. The increase in species richness with fire in 2002 suggests that the regrowth of nutritious vegetation outweighed the temporary removal of plant biomass (Van de Vijver et al. 1999). Fire may shift communities toward a diversity peak when burns are neither too frequent nor too extensive. This is consistent with the idea of intermediate disturbance resetting vegetation resources and increasing habitat heterogeneity.

### 4.2 Ungulate Species Richness Peaks Within About 1–3 km From Water

Our results suggest that areas within 1–3 km from water sources, where ungulate species richness peaked, should be prioritized for protection. With drought, we expected peaks in diversity and biomass to shift closer to water sources. However, the depletion of vegetation resources around these sources may reverse this trend. Water proximity can become a disturbance surrogate when frequent grazing and trampling by ungulates moving to and from water (Kanga et al. 2013) degrade forage. This can become more relevant when both wild and domestic ungulates need to access water on pastoral lands in a drought year. Increased predation risk near water (de Boer et al. 2010) could further depress ungulate species richness and biomass near water. Thus, regulating livestock access and reducing barriers are essential to maintaining forage and the value of areas near water.

### 4.3 Green Grass Is Most Important for Ungulate Biomass in a Drought Year

The availability of green grass, indexed by grass color, emerges as the most important predictor of reducing ungulate biomass loss in a drought year. This ranks forage greenness as a primary lever for drought resilience. The remarkable increase in ungulate biomass with grass color in the Mara Reserve in the drought year may reflect the relationship between nutrient content and color (Ryan et al. 2012). The peak in ungulate biomass at intermediate to high grass color scores under normal rainfall supports the prediction that ungulate biomass peaks at higher resource levels during drought than during normal rainfall. The lower peaks in ungulate species richness than in biomass at intermediate to high grass color scores in both years also support the prediction that biomass depends more on resources than diversity does. Wildebeest and zebra, which are major contributors to ungulate biomass, likely aggregated as pure grazers (Estes 2012) on green grass. In the drought year, these species contributed 50% more to total ungulate biomass when grass color exceeded a score of 7.5 in the Mara Reserve. Based on the humped‐back model, green grass may shift the system toward biomass rather than diversity maxima along the resources axis.

Although grass cover appeared less important than color for ungulate biomass, intermediate to high levels of grass cover were optimal for ungulate diversity and biomass. The peak in species richness at medium to high levels of grass cover aligns with the prediction that diversity peaks at intermediate levels of resources. But the comparatively stable ungulate biomass at higher levels of grass cover does not confirm the prediction that biomass peaks at higher levels of resources than diversity. This pattern may also reflect a detectability bias, whereby individuals are overlooked in high cover. Thus, interpretations should consider observation constraints. Nevertheless, these results suggest that most species avoid low and pure grass cover except in drought years. This emphasizes the importance of maintaining heterogeneous patches and avoiding the conversion of landscape into uniform grass areas.

Grass height shows how changes in ungulate diversity and biomass can differ with the same predictor depending on rainfall. Under normal rainfall conditions, tall grass that is hard to digest supported fewer ungulates and should be avoided. However, grass height was often an inconsistent predictor of ungulate diversity and biomass. This may be due to imperfect detection if animals are missed in tall grass. The decrease in raw species richness with increasing grass height in 2002 does not support the prediction that diversity peaks at intermediate levels of resources. But the less steep decrease in raw species richness with increasing grass height in the drought year partly supports the prediction that diversity peaks at higher levels of resources following a drought. In dry conditions, species such as zebra can become less selective and eat more tall grass than in wet conditions (Owaga 1975; Kleynhans et al. 2011). Height can therefore be treated as a modifier of accessibility and diet choice conditional on rainfall.

### 4.4 Shrubs and Trees May Increase Ungulate Diversity and Prevent Ungulate Biomass Loss in Drought Years

Woody vegetation can buffer drought to protect biomass while maintaining diversity. This aligns with the humped‐back trade‐off between resources, biomass, and diversity. Moderate shrub and tree cover can be crucial for ungulates during droughts, as indicated by the strong increase in ungulate biomass under shrub cover in the Mara Reserve and under tree cover on pastoral lands in 1999. Shrubs and trees can shield grasses from direct sunlight (Weltzin and Coughenour 1990) and provide food during water scarcity (Treydte et al. 2013). Increased predation risk in dense or tall shrubs (Valeix et al. 2009) may, however, imply nonlinear optimum rather than a monotonic benefit. Uncertainty from kriging may have biased predictions. The association between the observed and estimated tree cover in 1999 and shrub cover in both years was weaker than for other vegetation variables. These predictors should thus be treated cautiously.

The smaller increase in evenness‐adjusted diversity measures compared to biomass as shrub cover increased in the Mara Reserve in the drought year supports the prediction that biomass peaks at higher resource levels than diversity. The decrease in raw species richness with increasing shrub and tree cover in the reserve in the normal rainfall year may, however, suggest that more than 10%–20% shrub or tree cover could reduce the number of species adapted to more open savanna.

The increase in ungulate biomass relative to the peak in species richness at 30% tree cover on pastoral lands in 1999 suggests a resource‐skewed biomass maximum. Shrubs under 2 m and trees under 4 m tall may support higher ungulate diversity and biomass because they provide reachable browse. Yet ungulate biomass also peaked under large trees in the Mara Reserve in 2002. Green woody vegetation may signal the presence of water and nutrients, attracting diverse ungulates at medium to high shrub or tree color values.

Overall, woody vegetation illustrates a central management tension. While increased woody cover can offset the loss of ungulate biomass caused by drought, excessive expansion of woody vegetation can reduce ungulate richness. Maintaining this balance requires managing fire, grazing, and elephant populations (Dublin 1995; Dublin et al. 1990).

### 4.5 Ungulate Diversity and Biomass Peak on Low Slopes and Low to Intermediate Elevational Ranges

Our results suggest that low slopes and low‐to‐moderate elevations are ideal for ungulate diversity and biomass, although ungulates seemed less affected by changes in elevation in the Mara Reserve. The shift in peak ungulate biomass from low‐elevation areas in 2002 to mid‐elevation pastoral lands after the 1999 drought suggests that, during that time, ungulates sought shelter from the sun on tree‐ and shrub‐covered hillsides. These areas may offer more resources by shielding green grass from direct sunlight. Livestock also move to higher elevations for this reason during the dry season.

### 4.6 Rainfall and Carnivores Are Less Consistent Predictors of Ungulate Diversity and Biomass Than Other Environmental or Anthropogenic Variables

Rainfall and carnivores appear less consistent predictors than several variables related to vegetation, water and human use. This is not because these variables are unimportant, but rather their effects may be mediated through proximate mechanisms represented in the predictor set. Although biomass increases with rainfall on a continental scale (Coe et al. 1976), this relationship may not transfer directly to local landscapes (Bierhoff et al. 2024). Predators such as lions ( *Panthera leo* ) and hyenas ( *Crocuta crocuta* ) prey on diverse ungulates (Sinclair et al. 2003). Yet our results suggest that human activities, vegetation composition and water availability more consistently shape the community template on which predation operates. In practical terms, managers can more directly alter human use and its associated conditions than they can change rainfall or predator behavior. This makes human use and habitat levers both defensible and feasible.

### 4.7 Conservation Implications

The integrated disturbance‐resource synthesis yields a list of conservation and pastoral management priorities. These priorities include regulating settlement density and livestock composition, ensuring access to water and drought refuges, and maintaining heterogeneous grass and woody structures at intermediate disturbance levels. Ungulate diversity peaks on pastoral lands with low densities of sheep and goats. These lands should provide ungulates with at least 1 km, and ideally 3–4 km, of free space from settlements. In 1999 and 2002, areas beyond 2 km from bomas covered 63% of pastoral lands, while areas beyond 3 km accounted for about 10% of pastoral lands in the Mara Ecosystem. Therefore, the establishment of one new boma per km^2^ every 10 years within a 5 km buffer around the Mara Reserve from 2001 to 2016 (Probert et al. 2019) shifts conditions away from the intermediate zone in which diversity and biomass can be sustained together.

Conservation strategies on pastoral lands must consider more than just ecology because fully protected areas displace people (Butt 2014). Designating smaller zones with housing, employment opportunities, and infrastructure may reduce land demand while improving service delivery (Alaci 2010). This could ease tensions between mobility‐based pastoral strategies and fixed settlement designs.

Since 2005–2006, wildlife conservancies have provided a governance model that aligns benefits with conservation outcomes. Managed by local communities and tourism investors, these conservancies appear effective in slowing, halting, or even reversing the decline of ungulate populations (Barnes and De Jager 1996; Lindsey et al. 2013; Ogutu et al. 2017). They support livelihoods and prevent land fragmentation through sales and subdivision (Bedelian 2014). As such, they support low settlement density, which our results identified as critical for sustaining ungulate diversity and biomass. Conservancies aim to sustain livestock production and regulate grazing through rotational systems embedded in grazing management plans (Bedelian 2014; Herrik et al. 2023; Imbo et al. 2025). In the Mara, however, sheep and goat densities increased at similar rates inside and outside conservancies (Bedelian and Ogutu 2017). Since our results suggest that sheep and goats reduce ungulate species richness, grazing governance should address small‐stock expansion to ensure that diversity gains persist.

Instead of socially infeasible blanket restrictions, conservation design should prioritize stocking numbers and composition, spatial zoning and seasonal access. Model outputs support a practical decision‐making framework. The initial priorities are important and manageable: occupied bomas, livestock (primarily sheep and goats), access to water within 2–3 km, and indicators of nutritious forage (grass color and, secondarily, cover). Second‐order priorities are feasible but conditional. These include fire regimes, woody cover and color, and infrastructure placement. Depending on the context, these can increase heterogeneity or degrade habitats. Landscapes with low slopes, up to 1700 masl that are located near rivers or water points within 2–3 km can serve as suitable conservation areas. This depends on unobstructed access to water free of blockages caused by livestock, fences, people, settlements, or other barriers.

Conservancies implement a form of land‐use zoning that incorporates identified ecological thresholds. They designate areas for settlements, livestock grazing, and tourism, as well as core conservation zones where grazing is restricted (Bedelian 2014). This type of spatial planning translates ecological targets into feasible land‐use configurations by concentrating settlements into clusters and expanding settlement‐free areas. Involving landowners in this process ensures compliance and equitable outcomes (Bedelian et al. 2024), aligning ecological objectives with pastoral incentives. The challenge is not to exclude pastoral use but to organize it.

To maintain high species richness of ungulates and minimize biomass loss, especially in drought years, the grass on pastoral lands should be kept short. Trees should cover 20%–35% of the land, and shrubs should be less than 2 m tall. Protected areas with high grass cover can support high biomass dominated by grazers, such as wildebeest and zebra. To promote more balanced ungulate communities, protected areas should have about 65% green and partly tall grass, as well as 10%–20% green shrubs or trees. More woody vegetation can buffer drought losses, but it supports fewer species. In combination with rainfall and elephants (Dublin 1995; Dublin et al. 1990), fire management can control the growth of woody vegetation and increase ungulate richness. Fire should be applied judiciously because its effects differ among species (Anderson et al. 2020). Grazing species richness increases more than browser richness with fire (Klop and van Goethem 2008; Klop and Prins 2008).

## 5 Conclusions

Decisions regarding ungulate management should consider the disturbance‐resource continuum. High diversity requires intermediate disturbance and heterogeneous resources, whereas high biomass requires lower disturbance and greater resource availability, especially under drought. Distances of 1–2 km from bomas can support ungulate diversity on pastoral lands, while distances of 3–4 km can support both diversity and biomass. High shrub or tree cover can reduce drought‐driven loss of ungulate biomass, but cover exceeding 20%–30% can reduce species richness, consistent with a nonlinear optimum. Intermediate elevational ranges on pastoral lands may support high ungulate species richness and biomass in drought years. In contrast, lower elevational ranges may support higher biomass in years with normal rainfall. These findings suggest incorporating variation in vegetation and climate associated with changes in elevation into grazing and protection governance. Model outputs can help integrate these predictors into policy levers such as settlement and livestock governance, water availability, and fire and woody vegetation management, while considering socioeconomic constraints. Aligning interventions with the most important and manageable predictors and designing governance that reduces tension between pastoral use and conservation can help managers mitigate the effects of drought, maintain heterogeneous landscapes and improve coexistence. Long‐term monitoring can confirm whether land‐use rules and incentives can reduce disturbance to an intermediate level, thereby sustaining ungulate diversity and biomass.

## Author Contributions

**Gundula S. Bartzke:** conceptualization (equal), data curation (equal), formal analysis (lead), investigation (equal), methodology (equal), visualization (lead), writing – original draft (lead), writing – review and editing (equal). **Joseph O. Ogutu:** conceptualization (equal), data curation (equal), formal analysis (supporting), funding acquisition (supporting), investigation (supporting), methodology (equal), project administration (equal), supervision (equal), writing – review and editing (lead). **Hans‐Peter Piepho:** conceptualization (supporting), formal analysis (supporting), funding acquisition (supporting), methodology (supporting), supervision (supporting), writing – review and editing (equal). **Claire Bedelian:** data curation (supporting), investigation (supporting), writing – review and editing (supporting). **Michael E. Rainy:** data curation (supporting), investigation (supporting), writing – review and editing (supporting). **Russel L. Kruska:** data curation (supporting), investigation (supporting), writing – review and editing (supporting). **Jeffrey S. Worden:** data curation (supporting), investigation (supporting), writing – review and editing (supporting). **Kamau Kimani:** data curation (supporting), investigation (supporting), writing – review and editing (supporting). **Michael J. McCartney:** data curation (supporting), investigation (supporting), writing – review and editing (supporting). **Leah Ng'ang'a:** data curation (supporting), investigation (supporting), writing – review and editing (supporting). **Jeniffer Kinoti:** data curation (supporting), investigation (supporting), writing – review and editing (supporting). **Evanson C. Njuguna:** data curation (supporting), investigation (supporting), writing – review and editing (supporting). **Cathleen J. Wilson:** conceptualization (supporting), data curation (supporting), investigation (supporting), supervision (supporting), writing – review and editing (supporting). **Richard Lamprey:** data curation (supporting), investigation (supporting), writing – review and editing (supporting). **N. Thompson Hobbs:** funding acquisition (supporting), writing – review and editing (supporting). **Robin S. Reid:** conceptualization (equal), funding acquisition (lead), investigation (equal), methodology (equal), project administration (equal), supervision (equal), writing – review and editing (equal).

## Funding

This work was supported by Deutsche Forschungsgemeinschaft, 257734638, Belgian Government, DGIC BEL011, Directorate for Biological Sciences, DEB 0342820, 37 core donors of the International Livestock Research Institute.

## Conflicts of Interest

The authors declare no conflicts of interest.

## Supporting information


**Appendix S1:** ece373501‐sup‐0001‐AppendixS1.pdf.

**Appendix S2:** ece373501‐sup‐0002‐AppendixS2.pdf.

## Data Availability

The R code and data are archived on https://doi.org/10.5281/zenodo.17073514.
