# Supplementary material for: Balancing Savanna Ungulate Diversity and Biomass: Optimal Human Use, Landscape Features, and Vegetation Types Under Varying Rainfall and Land Use
Source: Ecol Evol. 2026 Apr 20;16(4):e73501. doi: 10.1002/ece3.73501 (PMC13095871; doi:10.1002/ece3.73501)
Supplement: Supplementary file 1 — Appendix S1: ece373501‐sup‐0001‐AppendixS1.pdf. [file ECE3-16-e73501-s002.pdf]

Appendix S1

Balancing Savanna Ungulate Diversity and Biomass: Optimal Human use, Landscape Features and Vegetation Types Under Varying Rainfall and Land Use

Ecology and Evolution

Gundula S. Bartzke, Joseph O. Ogutu, Hans-Peter Piepho, Claire Bedelian, Michael E. Rainy, Russel L. Kruska, Jeffrey S. Worden, Kamau Kimani, Michael J. McCartney, Leah Ng'ang'a, Jeniffer Kinoti, Evanson C. Njuguna, Cathleen J. Wilson, Richard Lamprey, N. Thompson Hobbs, Robin S. Reid

List of Figures

Figure S1 3
Figure S2 4
Figure S3 5
Figure S4 6
Figure S5 7
Figure S6 8
Figure S7 9
Figure S8 10
Figure S9 11
Figure S10 12
Figure S11 13
Figure S12 14
Figure S13 15
Figure S14 16
Figure S15 17

Figure S16 18
Figure S17 19
Figure S18 20
Figure S19 21
Figure S20 22
Figure S21 23
Figure S22 24
Figure S23 25
Figure S24 26
Figure S25 27
Figure S26 28

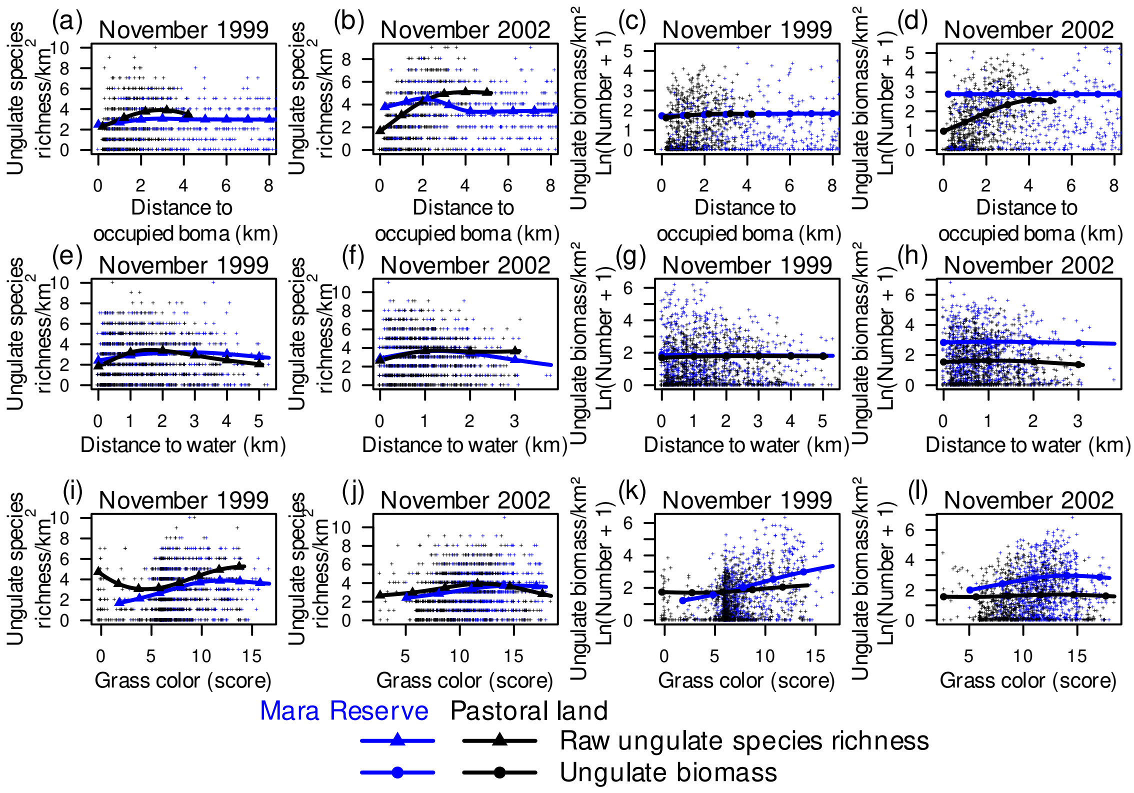

Figure S1: Raw species richness and log-transformed biomass of savanna ungulates in relation to distance to the nearest occupied boma (a-d), water (e-h) and grass color (i-l) in the Maasai Mara National Reserve (blue lines for predictions and blue pluses for observations) and adjacent pastoral lands (black lines for predictions and black pluses for observations) in Kenya in November of the 1999 drought year (a,c,e,g,i,k,m,o) and in November of the 2002 normal rainfall year (b,d,f,h,j,l,n,p). Predictions are truncated at 8 km from the nearest occupied boma.

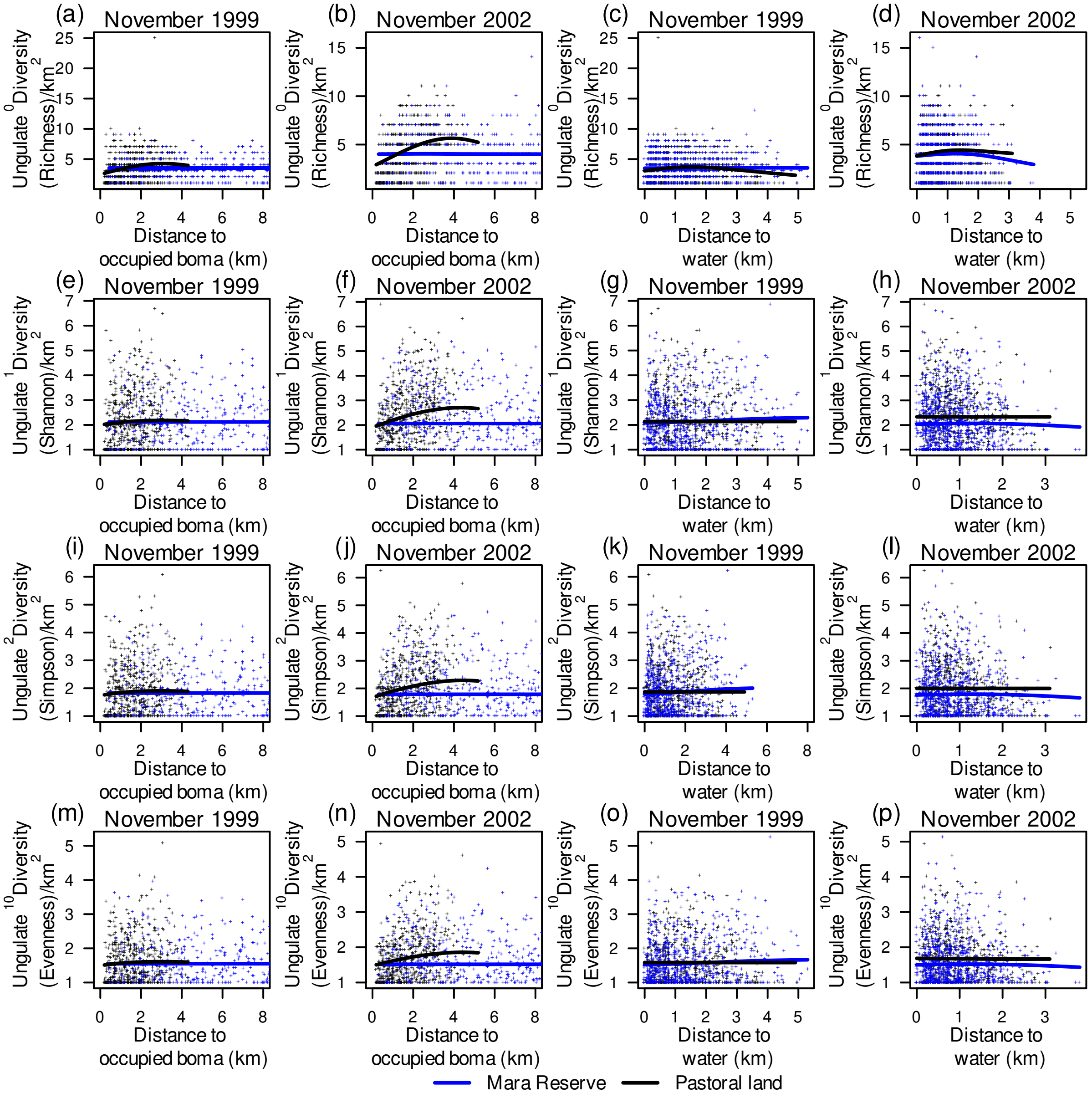

Figure S2: Ungulate diversity based on orders 0 (richness in a-d), 1 (Shannon in e-h), 2 (Simpson in i-l), 10 (species evenness in m-p) in relation to distance to occupied boma (a,b,e,f,i,j,m,n) and distance to water (c,d,g,h,k,l,o,p) in the Maasai Mara National Reserve (blue lines for predictions and blue pluses for observations) and adjacent pastoral lands (black lines for predictions and black pluses for observations) in Kenya in November of the 1999 drought year (a,c,e,g,i,k,m,o) and November of the 2002 normal rainfall year (b,d,f,h,j,l,n,p). Predictions are truncated at 8 km from the nearest occupied boma.

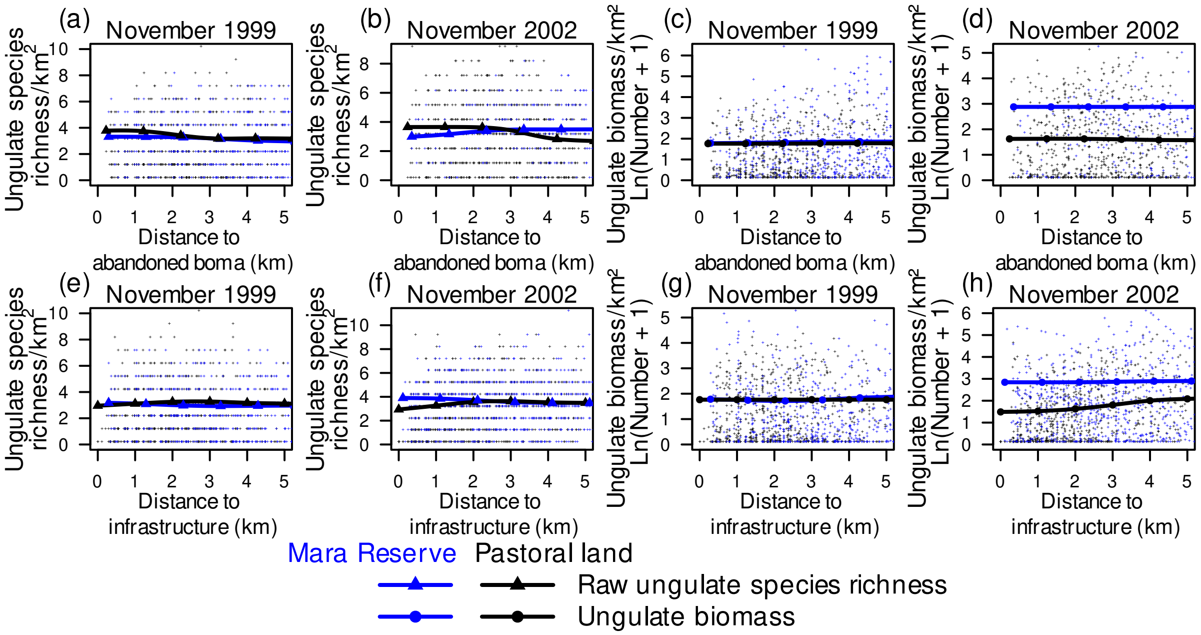

Figure S3: Raw species richness (lines with triangles in a,b,e,f) and log-transformed biomass (lines with dots in c,d,g,h) of savanna ungulates in relation to distance to the nearest abandoned boma (a-d) and infrastructure (e-h) in the Maasai Mara National Reserve (blue lines for predictions and blue pluses for observations) and adjacent pastoral lands (black lines for predictions and black pluses for observations) in Kenya in November of the 1999 drought year (a,c,e,g) and November of the 2002 normal rainfall year (b,d,f,h). Predictions are truncated at 5 km from the nearest abandoned boma or infrastructure.

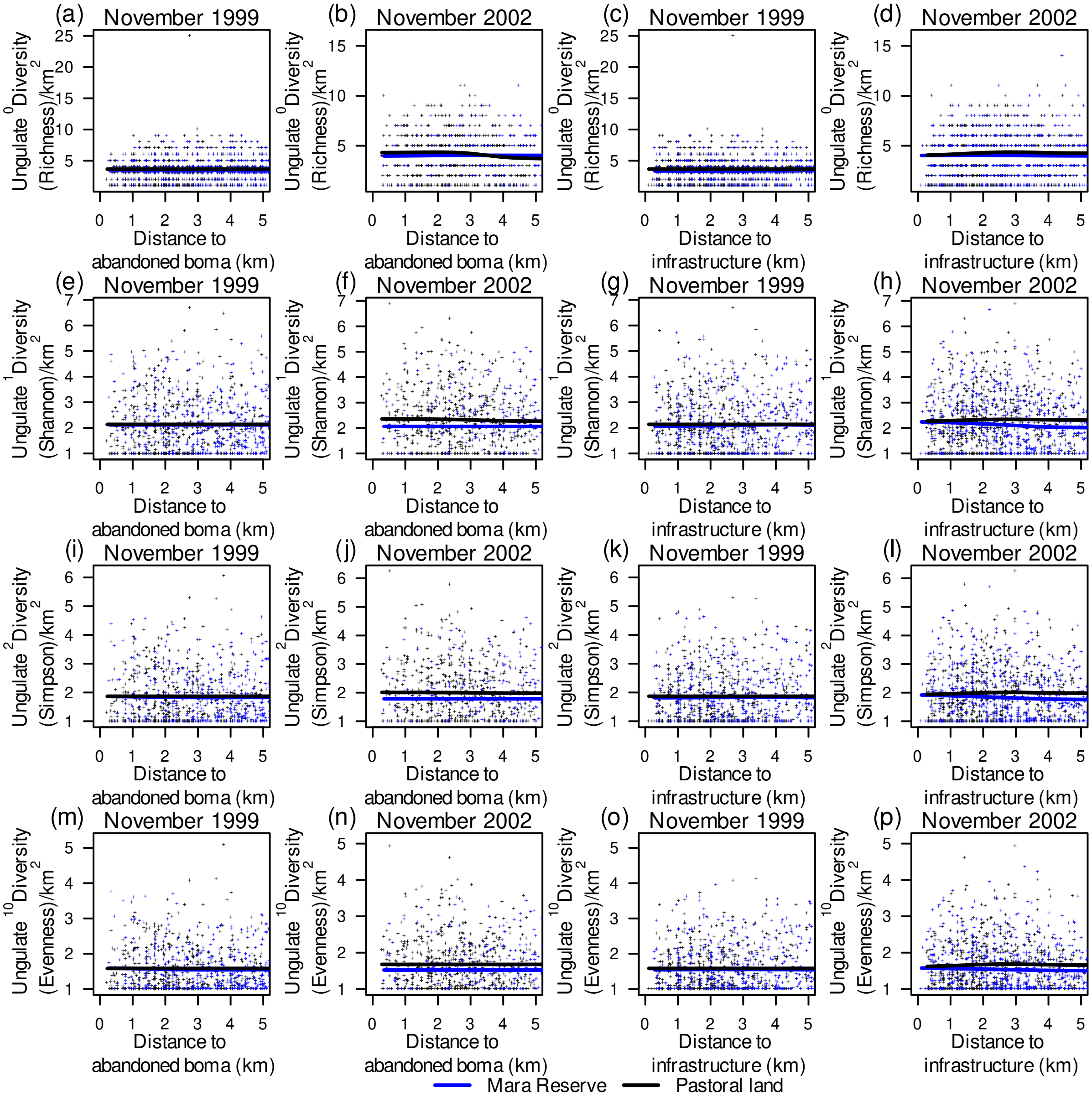

Figure S4: Ungulate diversity of orders 0 (richness: a-d), 1 (Shannon: e-h), 2 (Simpson: i-l) and 10 (species evenness: m-p) in relation to distance to the nearest abandoned boma (a,b,e,f,i,j,m,n) and infrastructure (c,d,g,h,k,l,o,p) in the Maasai Mara National Reserve (blue lines for predictions and blue pluses for observations) and adjacent pastoral lands (black lines for predictions and black pluses for observations) in Kenya in November of the 1999 drought year (a,c,e,g,i,k,m,o) and November of the 2002 normal rainfall year (b,d,f,h,j,l,n,p). Predictions are truncated at 5 km from the nearest abandonend boma or infrastructure.

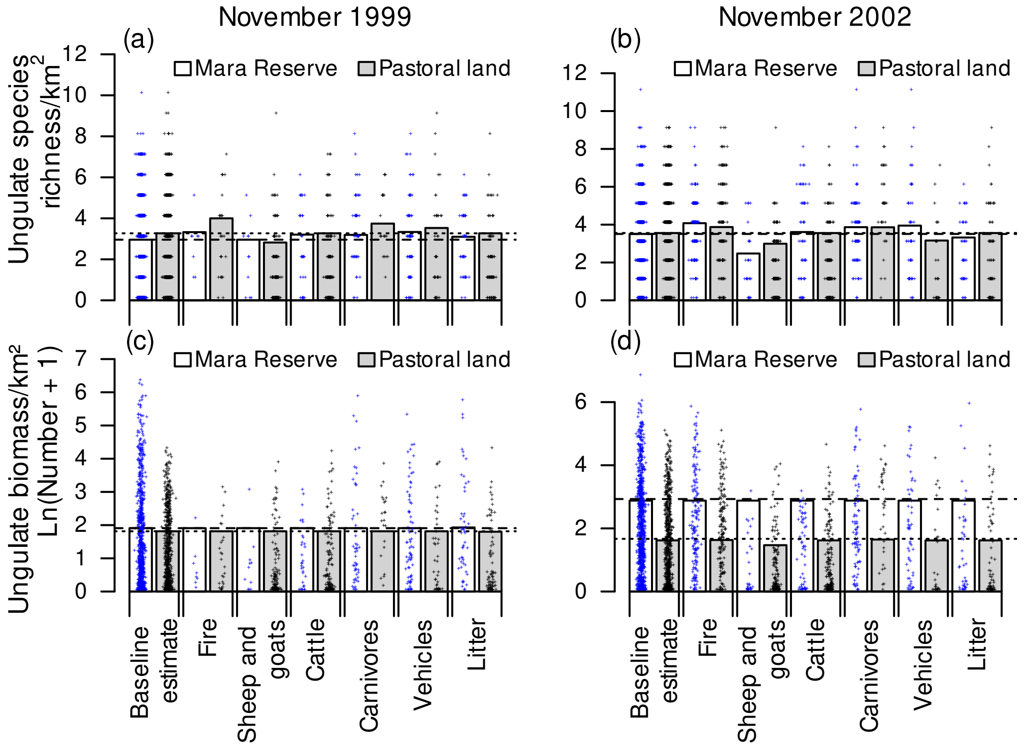

Figure S5: Raw species richness (a,b) and log-transformed biomass (c,d) of savanna ungulates in relation to fire, sheep and goats, cattle, carnivores, vehicles and litter in the Maasai Mara National Reserve (white bars for predictions and blue pluses for observations) and adjacent pastoral lands (grey bars for predictions and black pluses for observations) in November of the 1999 drought year (a,c) and November of the 2002 normal rainfall year (b,d).

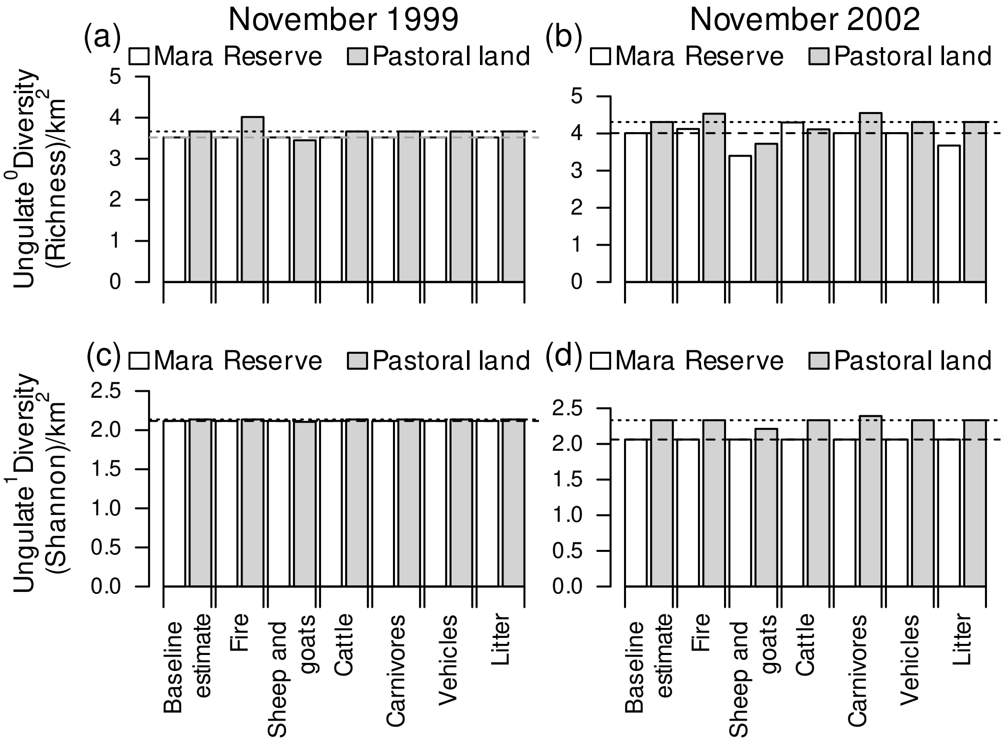

Figure S6: Effective number of species of savanna ungulates (a,b) based on diversity orders 0 (richness in a,b) and 1 (Shannon in c,d) in relation to fire, sheep and goats, cattle, carnivores, vehicles and litter in the Maasai Mara National Reserve (white bars) and adjacent pastoral lands (grey bars) in November of the 1999 drought year (a,c) and November of the 2002 normal rainfall year (b,d).

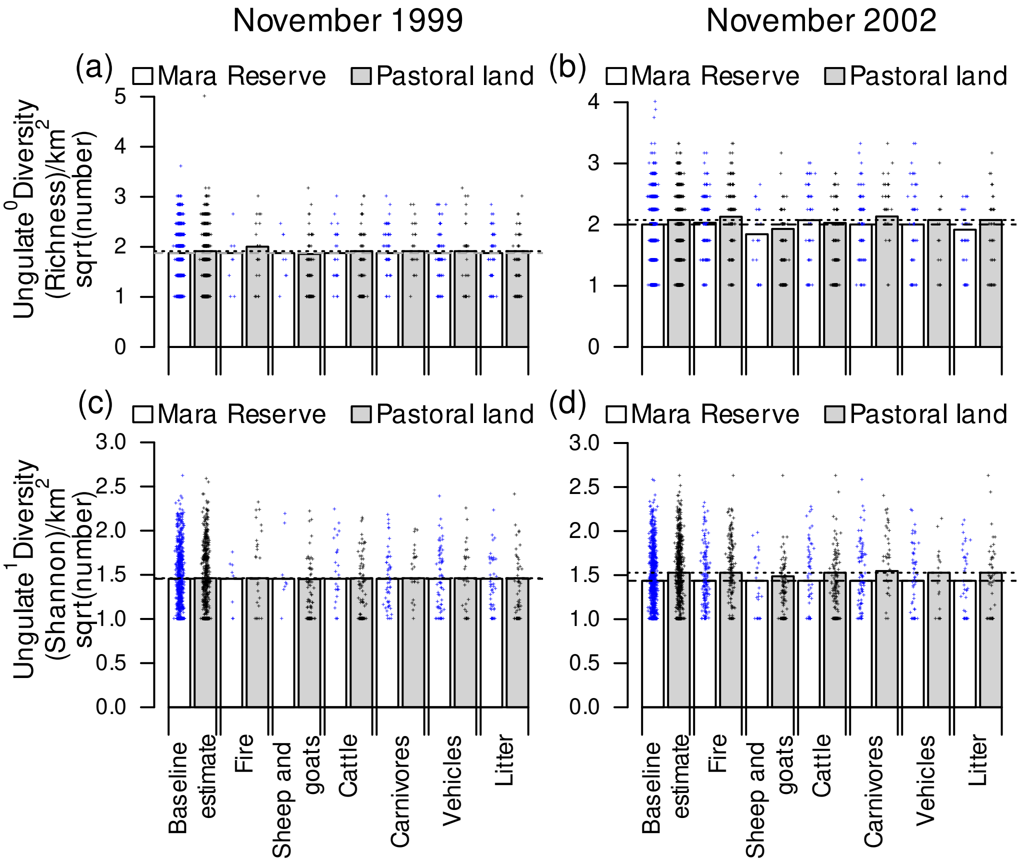

Figure S7: Square root of the effective number of species of savanna ungulates based on diversity orders 0 (species richness in a,b) and 1 (Shannon in c,d) in relation to fire, sheep and goats, cattle, carnivores, vehicles and litter in the Maasai Mara National Reserve (white bars for predictions and blue pluses for observations) and adjacent pastoral lands (grey bars for predictions and black pluses for observations) in November of the 1999 drought year (a,c) and November of the 2002 normal rainfall year (b,d).

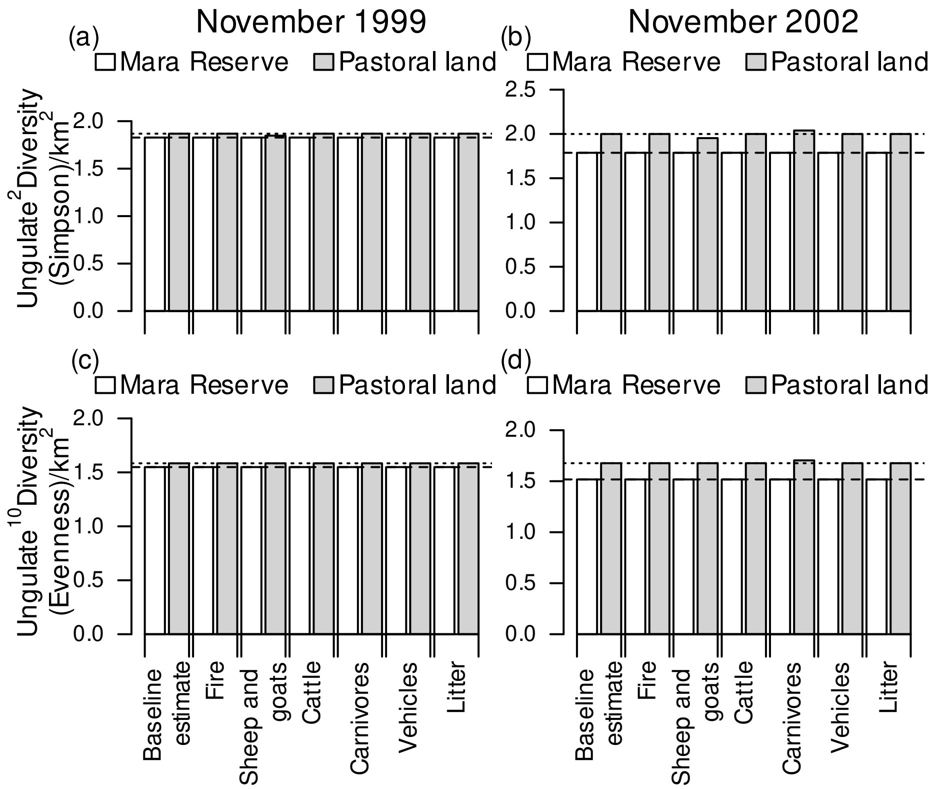

Figure S8: Effective number of species of savanna ungulates (a,b) based on diversity orders 2 (Simpson in a,b) and 10 (evenness in c,d) in relation to fire, sheep and goats, cattle, carnivores, vehicles and litter in the Maasai Mara National Reserve (white bars) and adjacent pastoral lands (grey bars) in November of the 1999 drought year (a,c) and November of the 2002 normal rainfall year (b,d).

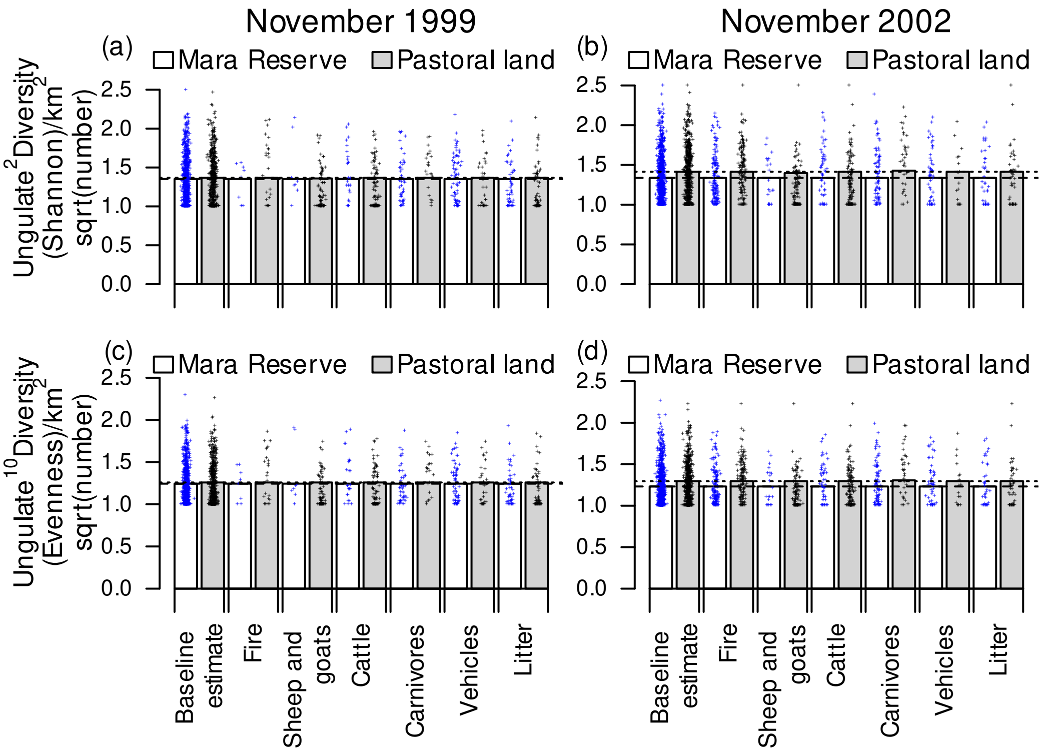

Figure S9: Square root of the effective number of species of savanna ungulates based on diversity orders 2 (Simpson in a,b) and 10 (evenness in c,d) in relation to fire, sheep and goats, cattle, carnivores, vehicles and litter in the Maasai Mara National Reserve (white bars for predictions and blue pluses for observations) and adjacent pastoral lands (grey bars for predictions and black pluses for observations) in November of the 1999 drought year (a,c) and November of the 2002 normal rainfall year (b,d).

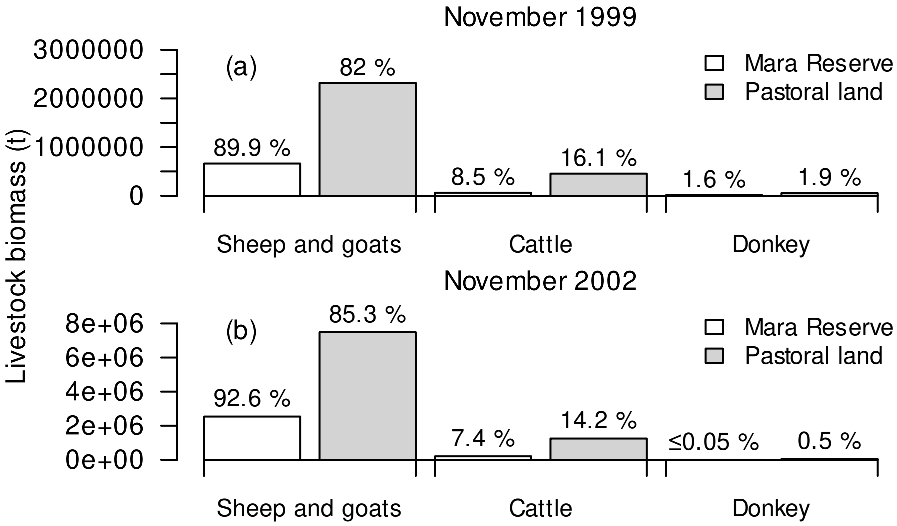

Figure S10: Contribution of sheep and goats, cattle and donkey to the total livestock biomass in November of the 1999 drought year (a) and November of the 2002 normal rainfall year (b) for the Maasai Mara National Reserve (white bars) and the adjacent pastoral lands (grey bars) in Kenya.

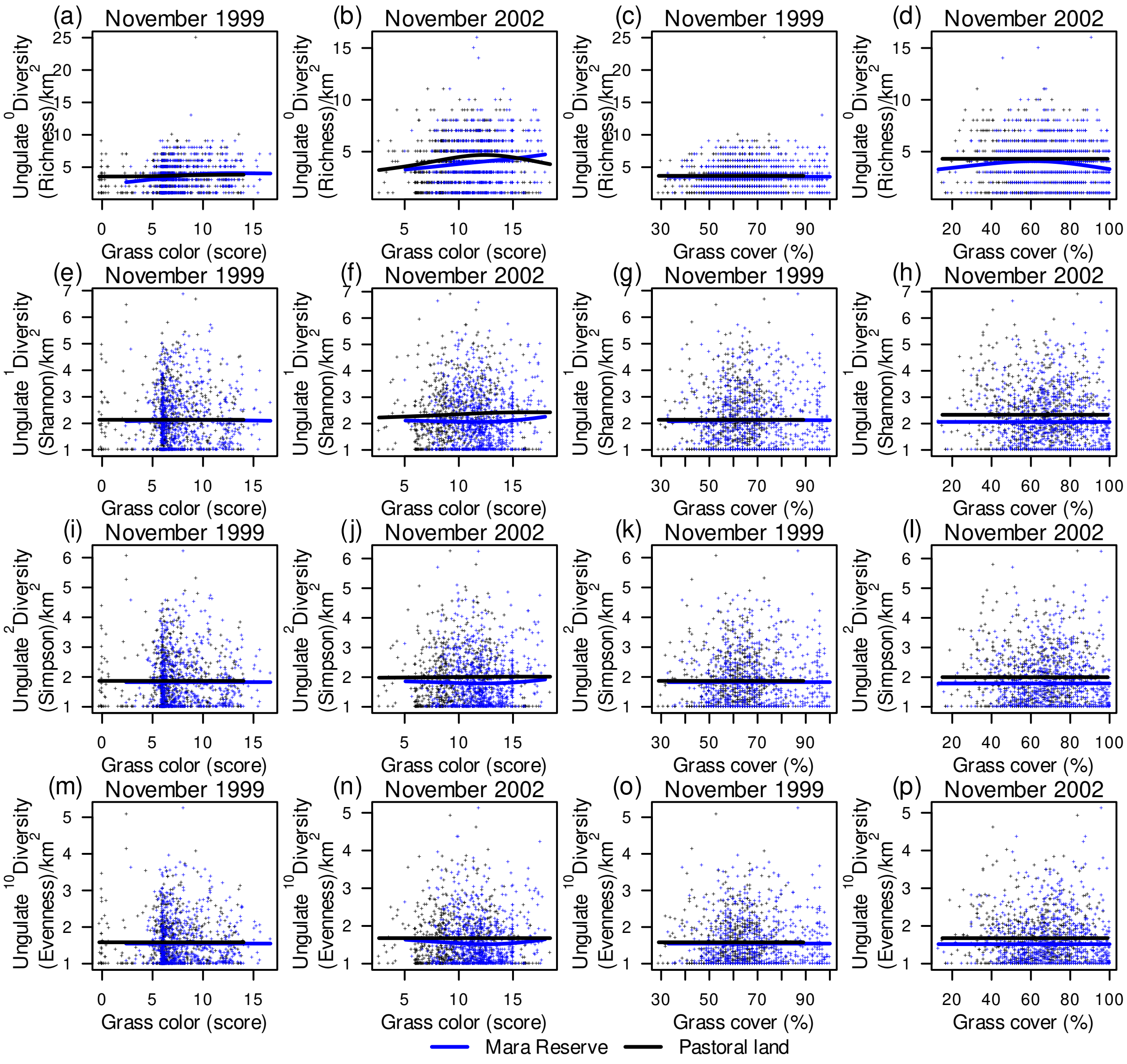

Figure S11: Ungulate diversity based on orders 0 (richness in a-d), 1 (Shannon in e-h), 2 (Simpson in i-l) and 10 (species evenness in m-p) in relation to grass color (a,b,e,f,i,j,m,n) and grass cover (c,d,g,h,k,l,o,p) in the Maasai Mara National Reserve (blue lines for predictions and blue pluses for observations) and adjacent pastoral lands (black lines for predictions and black pluses for observations) in Kenya in November of the 1999 drought year (a,c,e,g,i,k,m,o) and November of the 2002 normal rainfall year (b,d,f,h,j,l,n,p).

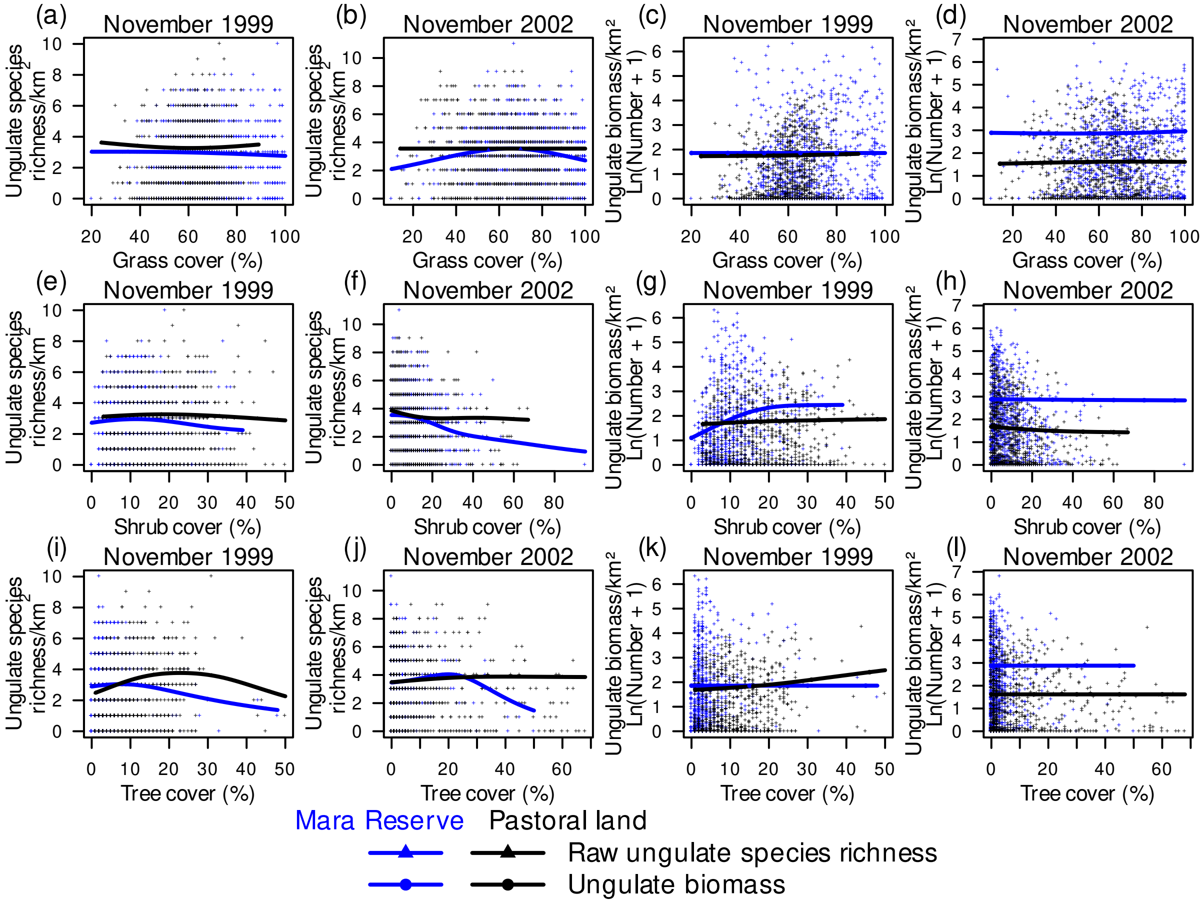

Figure S12: Raw species richness (continuous lines with triangles: a,b,e,f,i,j) and log-transformed biomass (continuous lines with dots: c,d,g,h,k,l) of savanna ungulates in relation to grass cover (a-d), shrub cover (e-h) or tree cover (i-l) in the Maasai Mara National Reserve (blue lines for predictions and blue pluses for observations) and adjacent pastoral lands (black lines for predictions and black pluses for observations) in Kenya in November of the 1999 drought year (a,c,e,g,i,k) and November of the 2002 normal rainfall year (b,d,f,h,j,l).

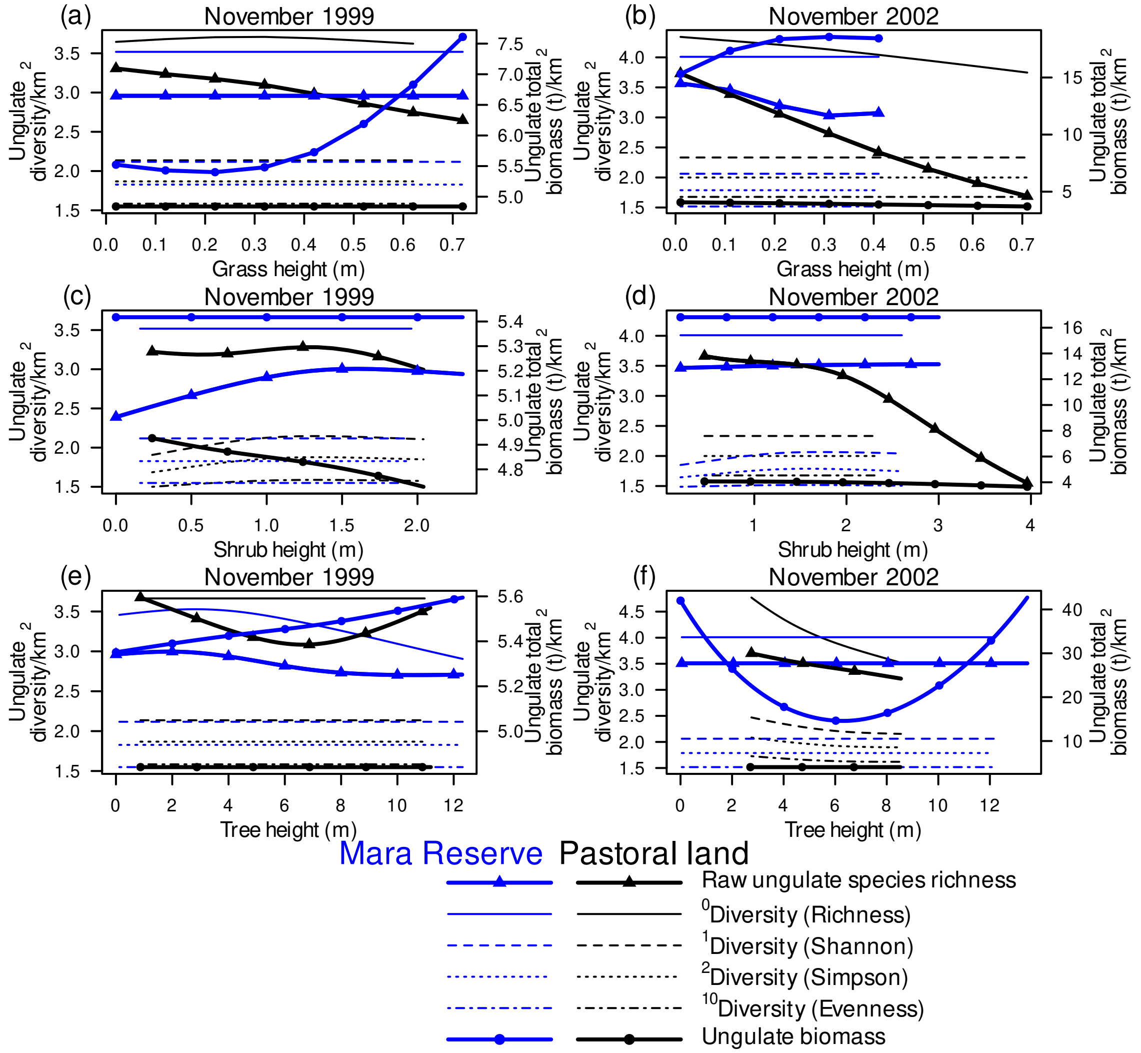

Figure S13: Ungulate diversity, expressed as the raw species richness (continuous thick lines with triangles), the bias-adjusted effective number of species based on diversity orders 0 (richness: continuous lines), 1 (Shannon: dashed lines), 2 (Simpson: dotted lines) and 10 (evenness: dashed-dotted lines), and biomass (continuous thick lines with dots) of savanna ungulates in relation to grass height (a,b), shrub height (c,d) or tree height (e,f) in the Maasai Mara National Reserve (blue lines) and adjacent pastoral lands (black lines) in Kenya in November of the 1999 drought year (a,c,e) and November of the 2002 normal rainfall year (b,d,f).

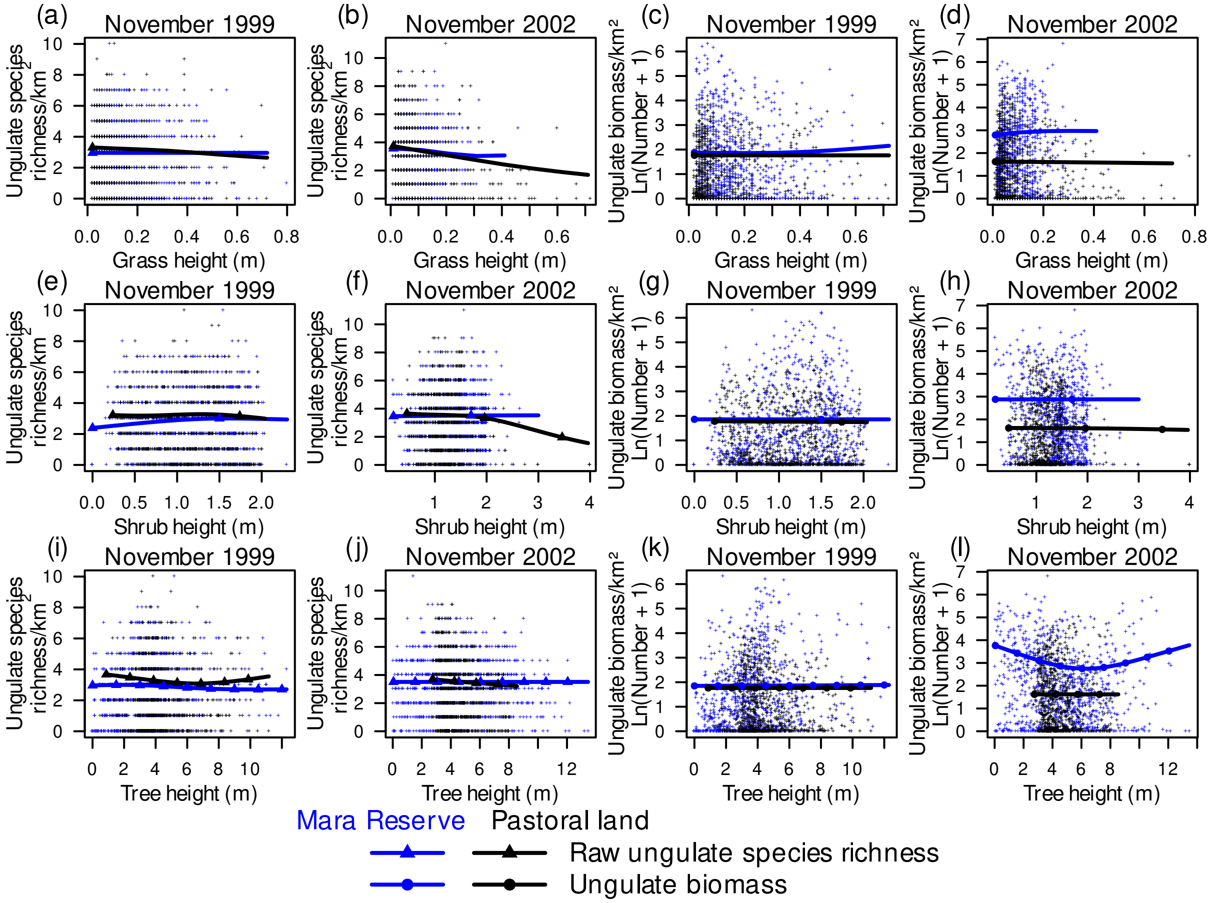

Figure S14: Raw species richness (continuous lines with triangles; a,b,e,f,i,j) and log-transformed biomass (continuous lines with dots; c,d,g,h,k,l) of savanna ungulates in relation to grass height (a-d), shrub height (e-h) or tree height (i-l) in the Maasai Mara National Reserve (blue lines for predictions and blue pluses for observations) and adjacent pastoral lands (black lines for predictions and black pluses for observations) in Kenya in the drought year of 1999 (a,c,e,g,i,k) and in 2002 (b,d,f,h,j,l).

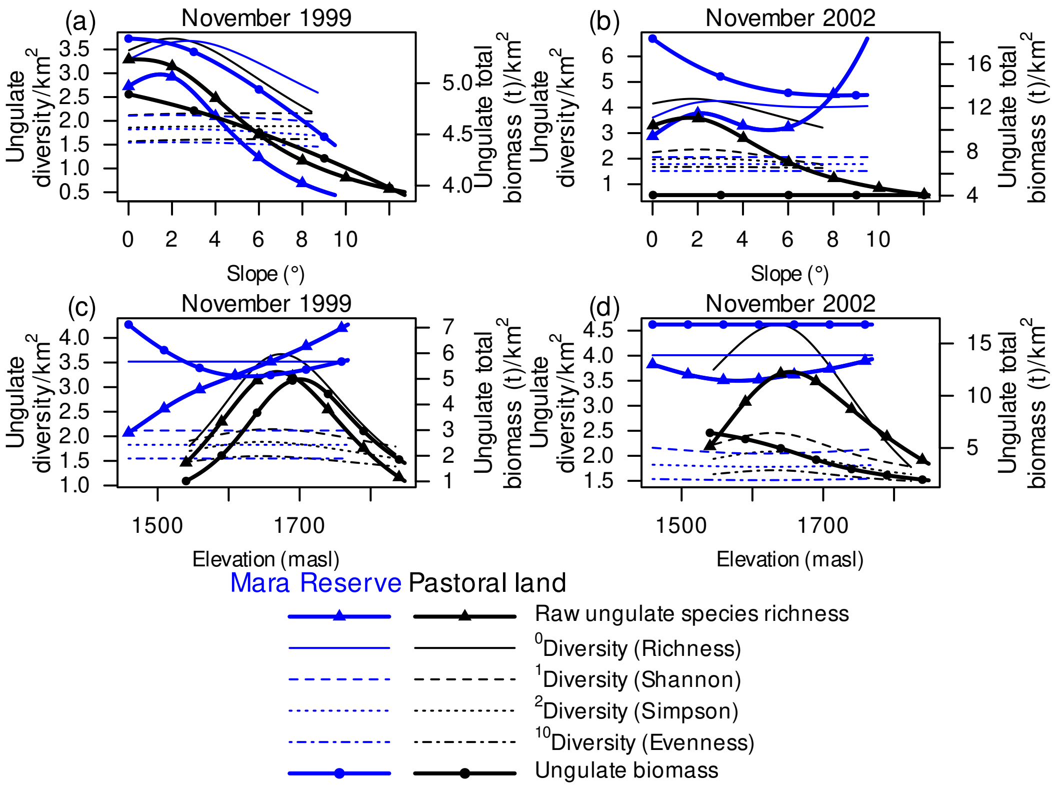

Figure S15: Ungulate diversity, expressed as the raw species richness (continuous thick lines with triangles), the bias-adjusted effective number of species based on diversity orders 0 (richness: continuous lines), 1 (Shannon: dashed lines), 2 (Simpson: dotted lines) and 10 (evenness: dashed-dotted lines), and biomass (continuous thick lines with dots) of savanna ungulates in relation to slope (a,b) and elevation (c,d) in the Maasai Mara National Reserve (blue lines) and adjacent pastoral lands (black lines) in Kenya in November of the 1999 drought year (a,c) and November of the 2002 normal rainfall year (b,d).

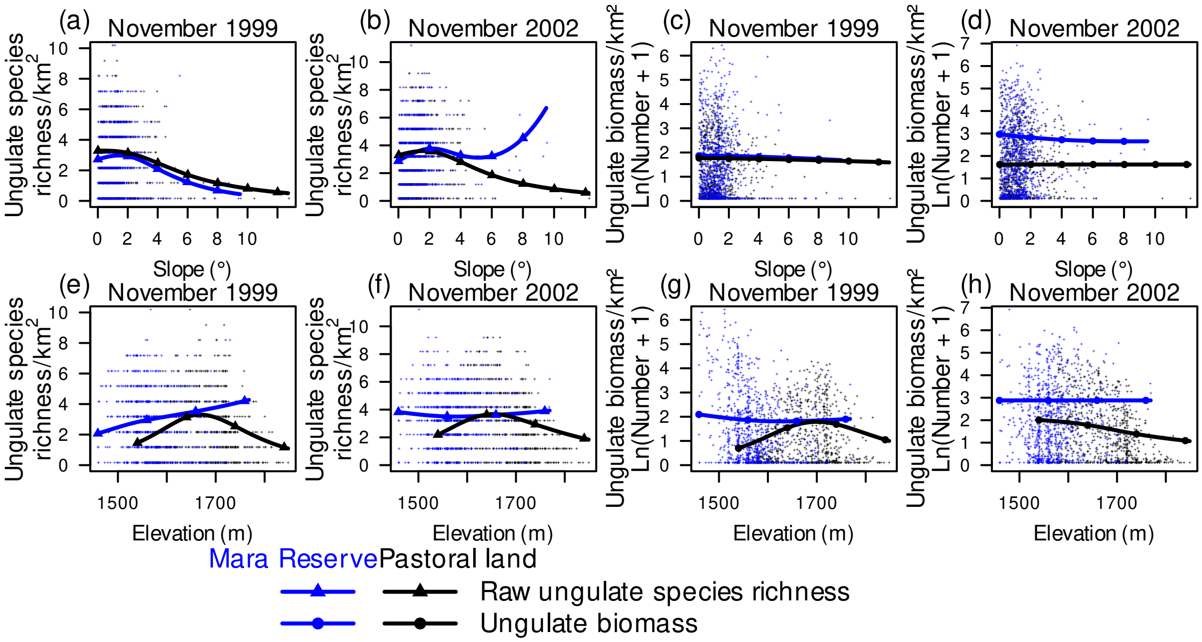

Figure S16: Raw species richness (continuous lines with triangles in a,b,e,f) and log-transformed biomass (continuous lines with dots in c,d,g,h) of savanna ungulates in relation to slope (a-d) and elevation (e-h) in the Maasai Mara National Reserve (blue lines for predictions and blue pluses for observations) and adjacent pastoral lands (black lines for predictions and black pluses for observations) in Kenya in November of the 1999 drought year (a,c,e,g) and November of the 2002 normal rainfall year (b,d,f,h).

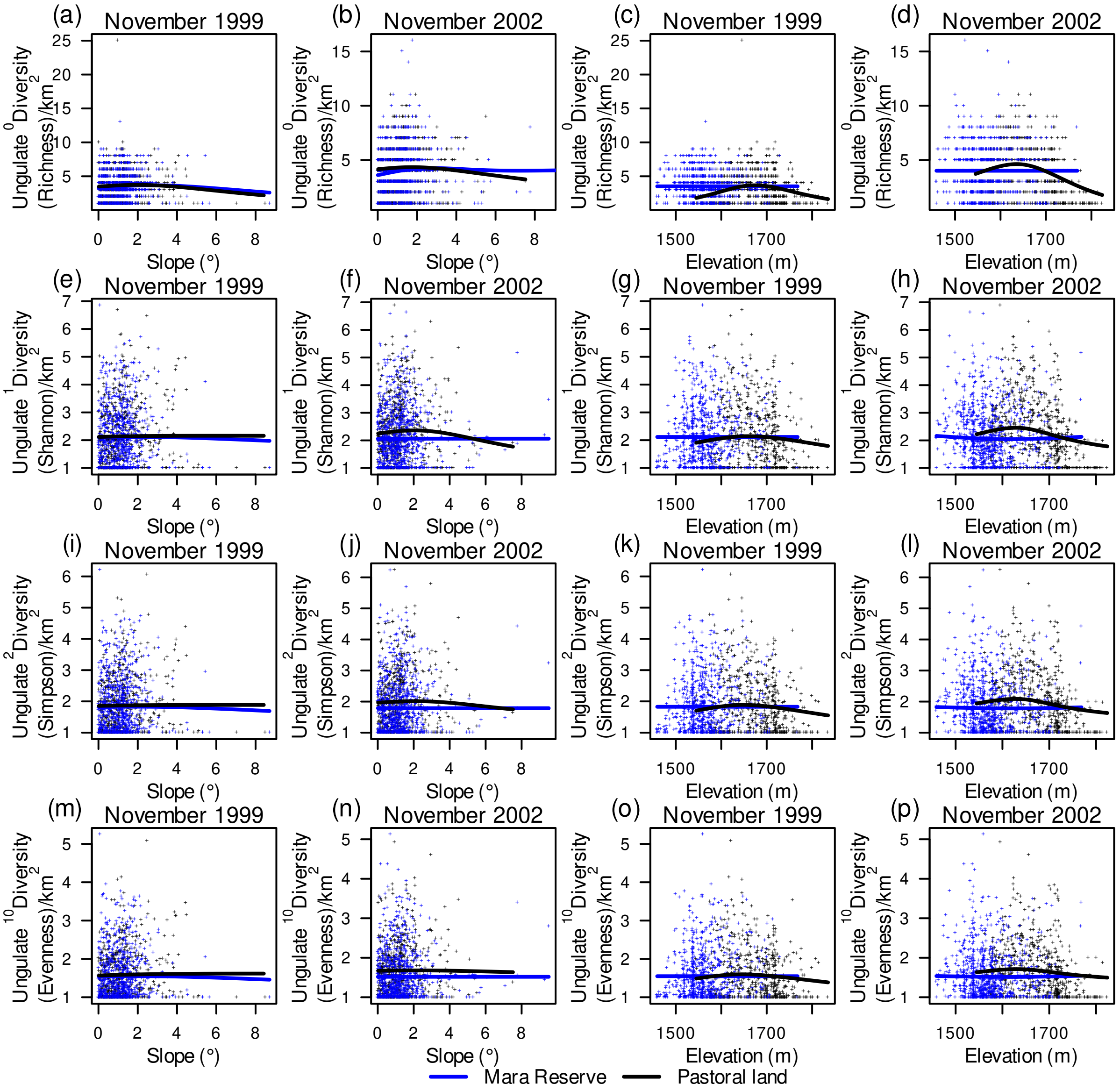

Figure S17: Ungulate diversity based on orders 0 (richness in a-d), 1 (Shannon in e-h), 2 (Simpson in i-l) and 10 (species evenness in m-p) in relation to slope (a,b,e,f,i,j,m,n) and elevation (c,d,g,h,k,l,o,p) in the Maasai Mara National Reserve (blue lines for predictions and blue pluses for observations) and adjacent pastoral lands (black lines for predictions and black pluses for observations) in Kenya in November of the 1999 drought year (a,c,e,g,i,k,m,o) and November of the 2002 normal rainfall year (b,d,f,h,j,l,n,p).

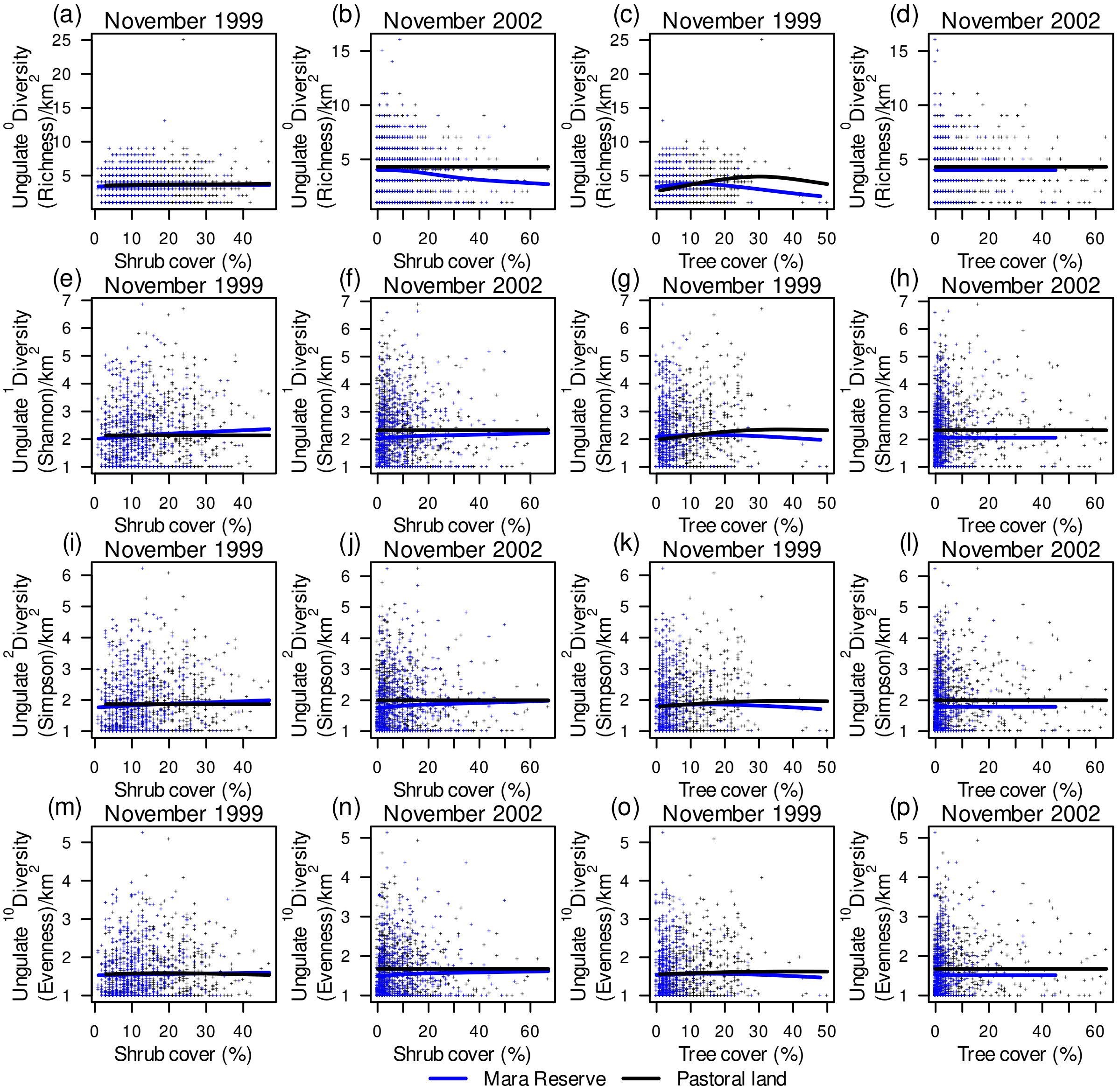

Figure S18: Ungulate diversity of orders 0 (richness: a-d), 1 (Shannon: e-h), 2 (Simpson: i-l) and 10 (species evenness: m-p) in relation to shrub cover (a,b,e,f,i,j,m,n) and tree cover (c,d,g,h,k,l,o,p) in the Maasai Mara National Reserve (blue lines for predictions and blue pluses for observations) and adjacent pastoral lands (black lines for predictions and black pluses for observations) in Kenya in November of the 1999 drought year (a,c,e,g,i,k,m,o) and in November of the 2002 normal rainfall year (b,d,f,h,j,l,n,p).

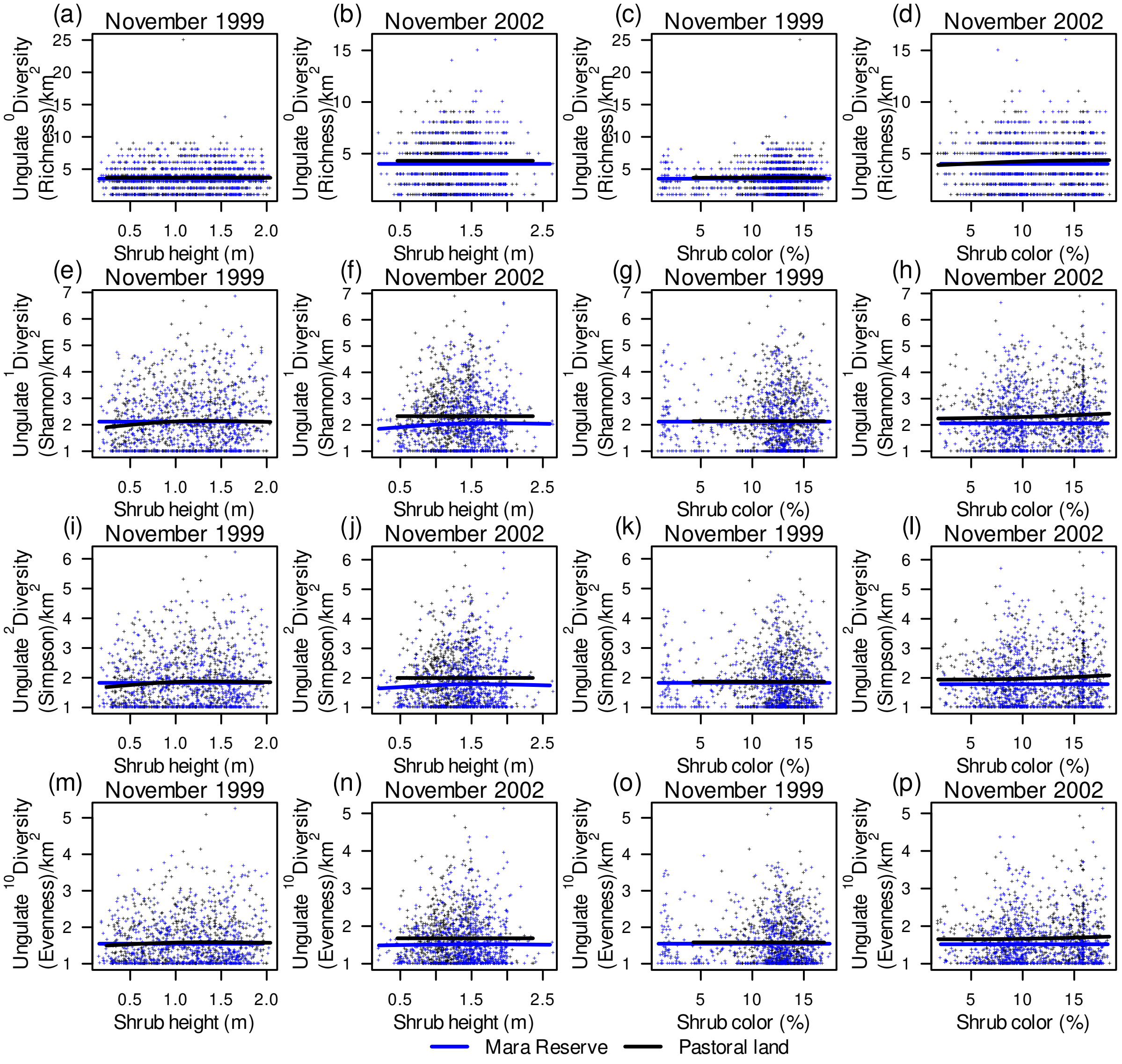

Figure S19: Ungulate diversity of orders 0 (richness: a-d), 1 (Shannon: e-h), 2 (Simpson: i-l) and 10 (species evenness: m-p) in relation to shrub height (a,b,e,f,i,j,m,n) and shrub color (c,d,g,h,k,l,o,p) in the Maasai Mara National Reserve (blue lines for predictions and blue pluses for observations) and adjacent pastoral lands (black lines for predictions and black pluses for observations) in Kenya in November of the 1999 drought year (a,c,e,g,i,k,m,o) and November of the 2002 normal rainfall year (b,d,f,h,j,l,n,p).

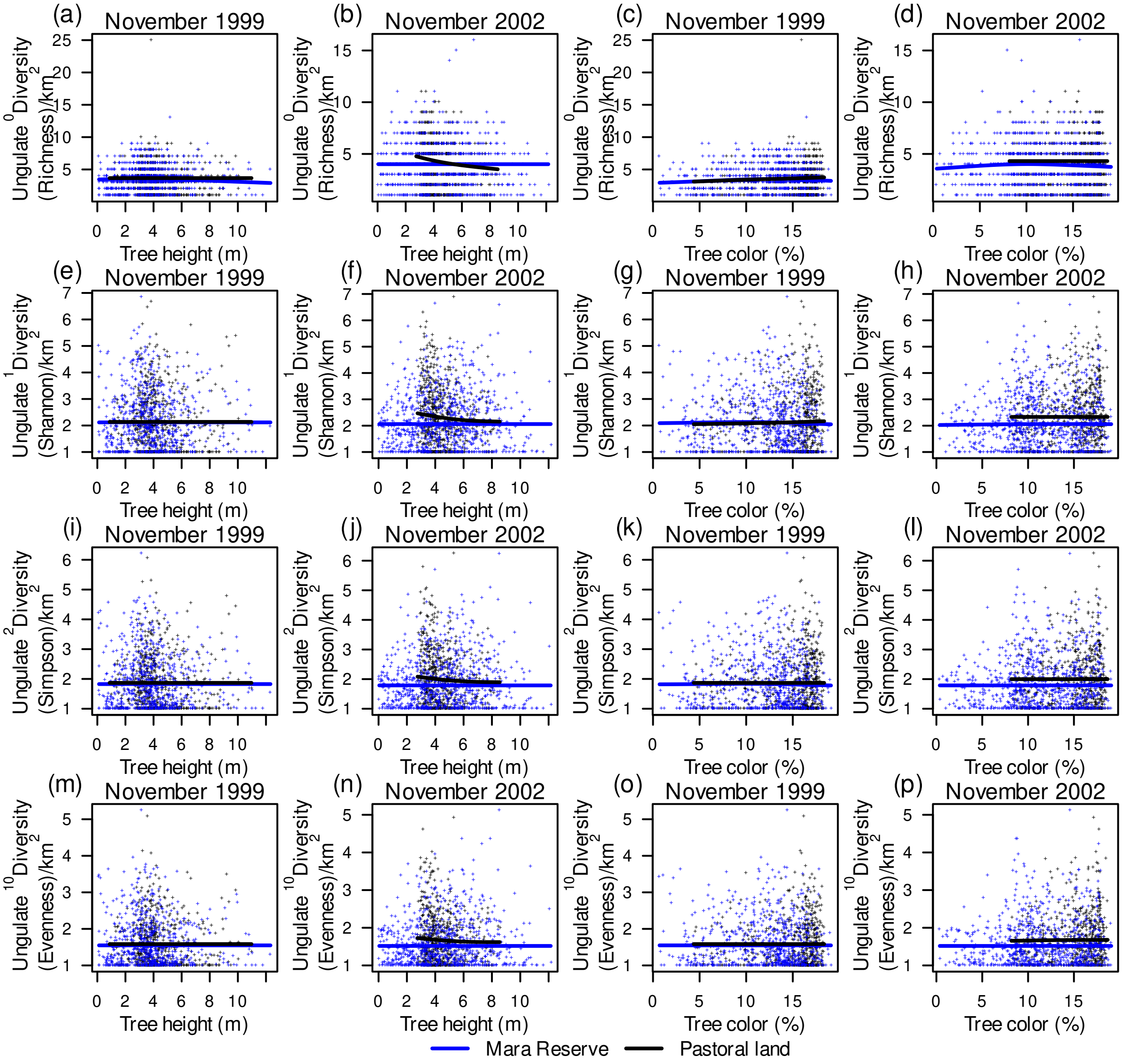

Figure S20: Ungulate diversity of orders 0 (richness: a-d), 1 (Shannon: e-h), 2 (Simpson: i-l) and 10 (species evenness: m-p) in relation to tree height (a,b,e,f,i,j,m,n) and tree color (c,d,g,h,k,l,o,p) in the Maasai Mara National Reserve (blue lines for predictions and blue pluses for observations) and adjacent pastoral lands (black lines for predictions and black pluses for observations) in Kenya in November of the 1999 drought year (a,c,e,g,i,k,m,o) and November of the 2002 normal rainfall year (b,d,f,h,j,l,n,p).

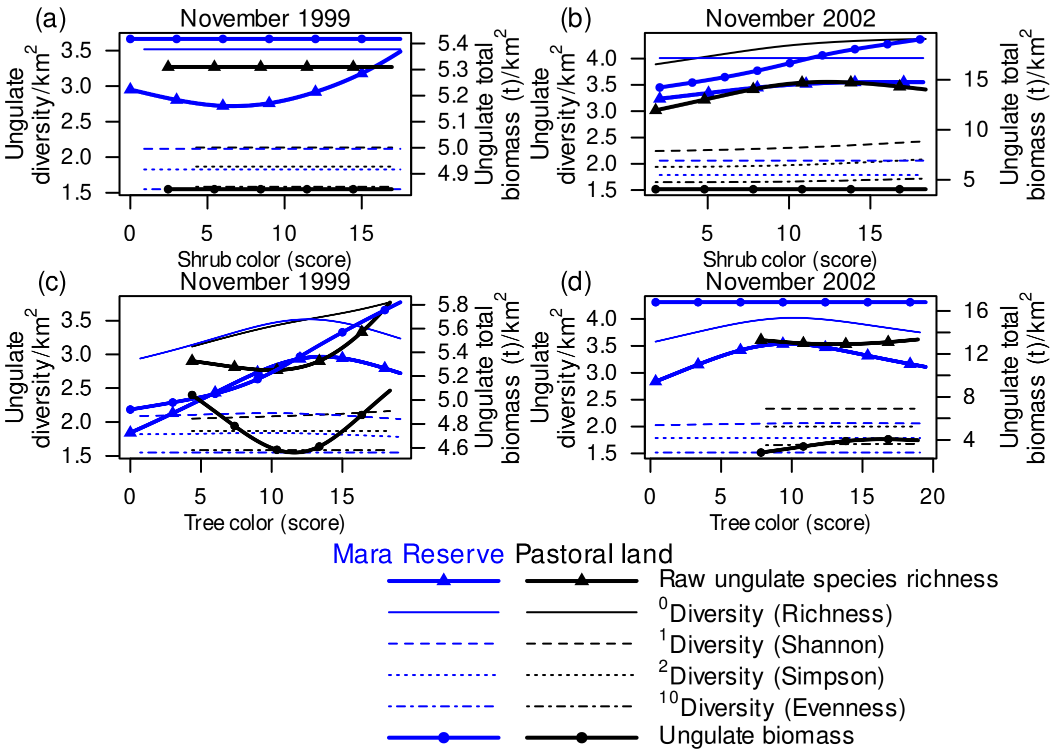

Figure S21: Ungulate diversity, expressed as the raw species richness (continuous thick lines with triangles), the bias-adjusted effective number of species based on diversity orders 0 (richness: continuous lines), 1 (Shannon: dashed lines), 2 (Simpson: dotted lines) and 10 (evenness: dashed-dotted lines), and biomass (continuous thick lines with dots) of savanna ungulates in relation to shrub color (a,b) or tree color (c,d) in the Maasai Mara National Reserve (blue lines) and adjacent pastoral lands (black lines) in Kenya in November of the 1999 drought year (a,c) and November of the 2002 normal rainfall year (b,d).

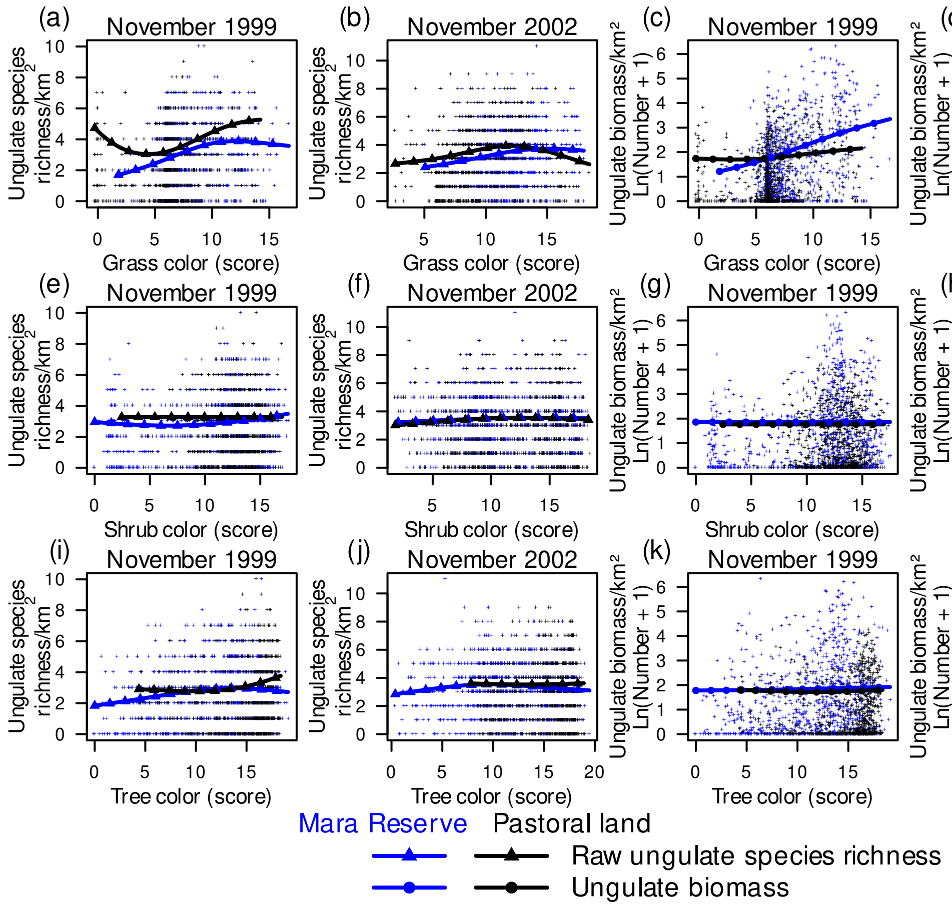

Figure S22: Raw species richness (continuous lines with triangles: a,b,e,f,i,j) and log-transformed biomass (continuous lines with dots: c,d,g,h,k,l) of savanna ungulates in relation to grass color (a-d), shrub color (e-h) or tree color (i-l) in the Maasai Mara National Reserve (blue lines for predictions and blue pluses for observations) and adjacent pastoral lands (black lines for predictions and black pluses for observations) in Kenya in November of the 1999 drought year (a,c,e,g,i,k) and November of the 2002 normal rainfall year (b,d,f,h,j,l).

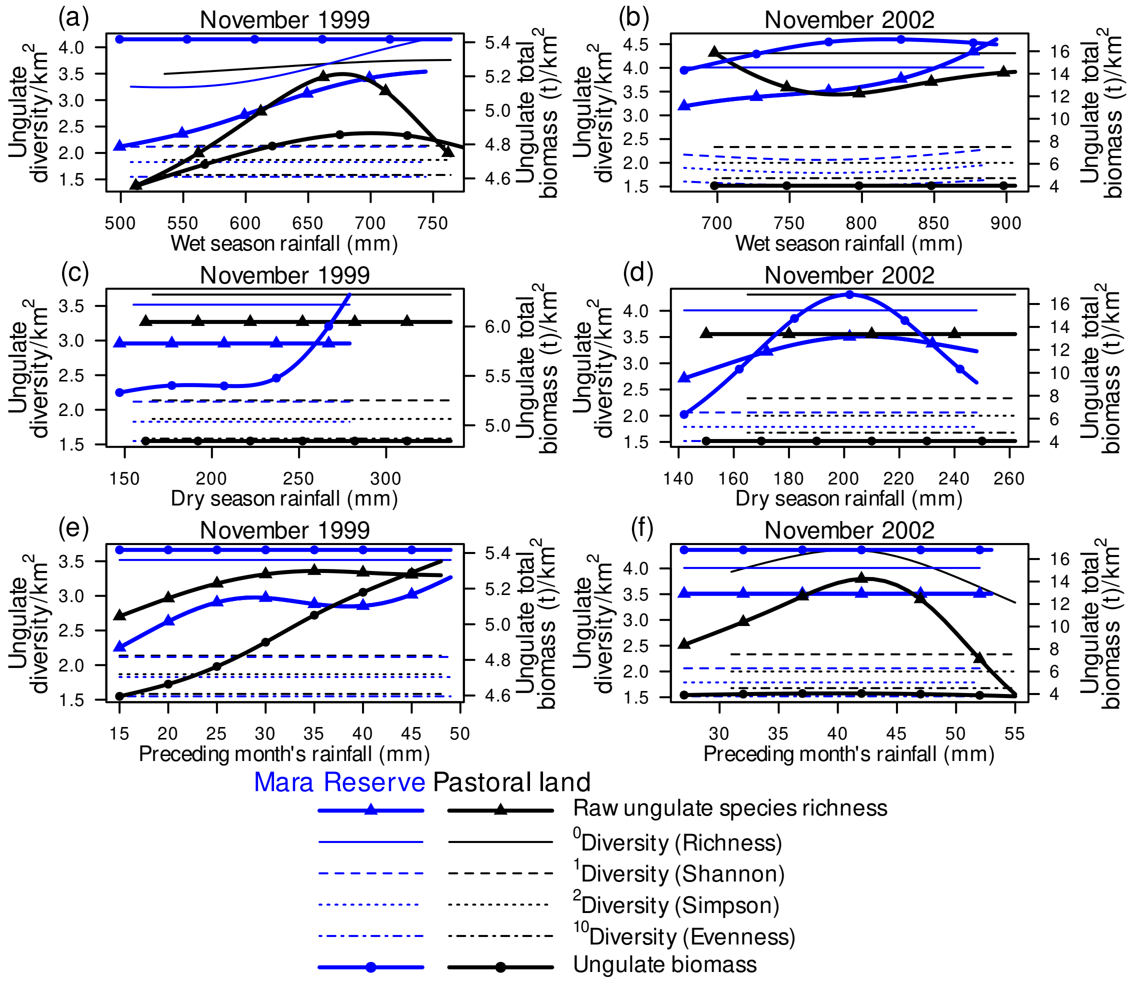

Figure S23: Ungulate diversity, expressed as the raw species richness (continuous thick lines with triangles), the bias-adjusted effective number of species based on diversity orders 0 (richness: continuous lines), 1 (Shannon: dashed lines), 2 (Simpson: dotted lines) and 10 (evenness: dashed-dotted lines), or biomass (continuous thick lines with dots) of savanna ungulates in relation to the wet season rainfall (a,b), dry season rainfall (c,d) or preceding month's rainfall (e,f) in the Maasai Mara National Reserve (blue lines) and adjacent pastoral lands (black lines) in Kenya in November of the 1999 drought year (a,c,e) and November of the 2002 normal rainfall year (b,d,f).

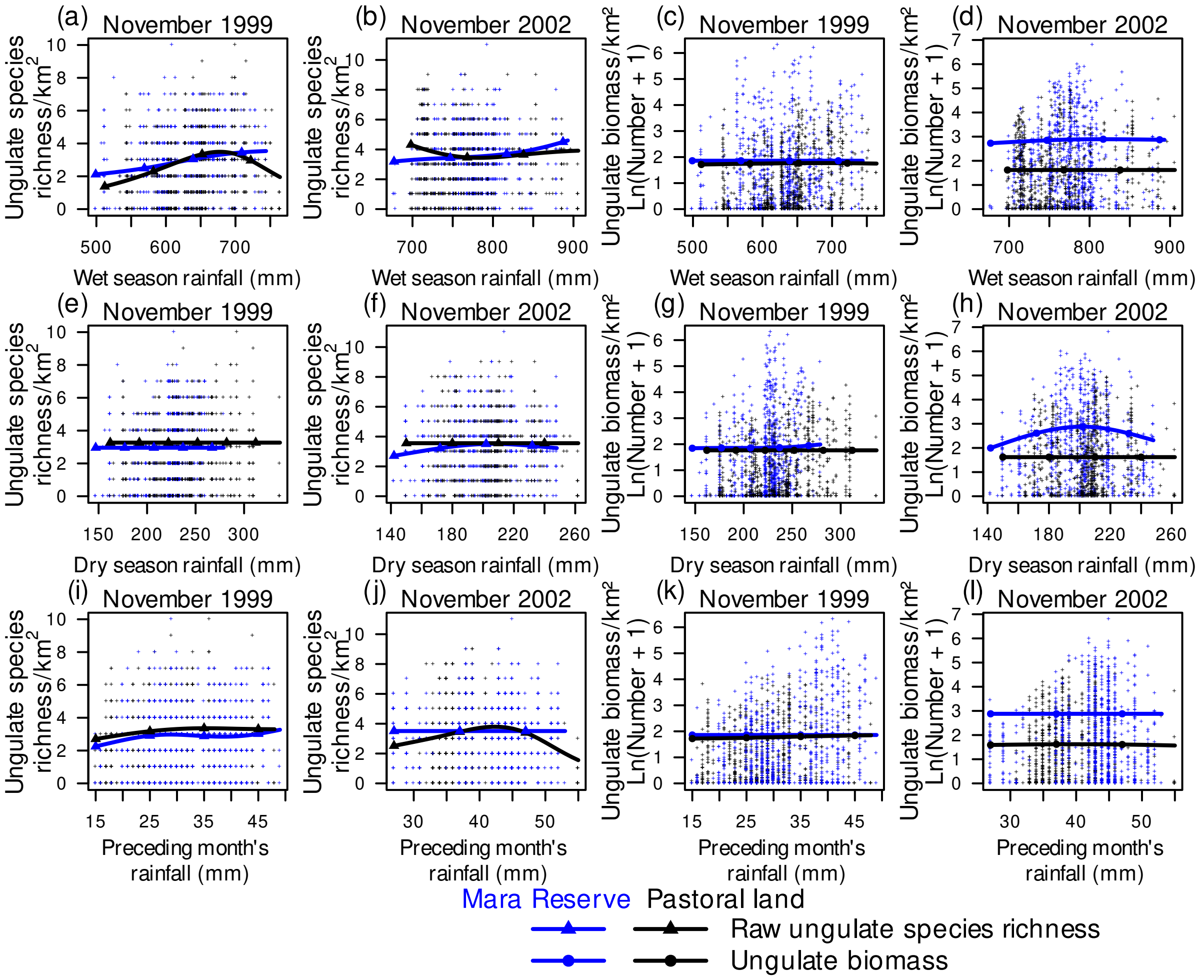

Figure S24: Raw species richness (continuous thick lines with triangles in a,b,e,f,i,j) or log-transformed biomass (continuous thick lines with dots in c,d,g,h,k,l) of savanna ungulates in relation to wet season rainfall (a-d), dry season rainfall (e-h) or monthly rainfall (i-l) in the Maasai Mara National Reserve (blue lines for predictions and blue pluses for observations) and adjacent pastoral lands (black lines for predictions and black pluses for observations) in Kenya in November of the 1999 drought year (a,c,e,g,i,k) and November of the 2002 normal rainfall year (b,d,f,h,j,l).

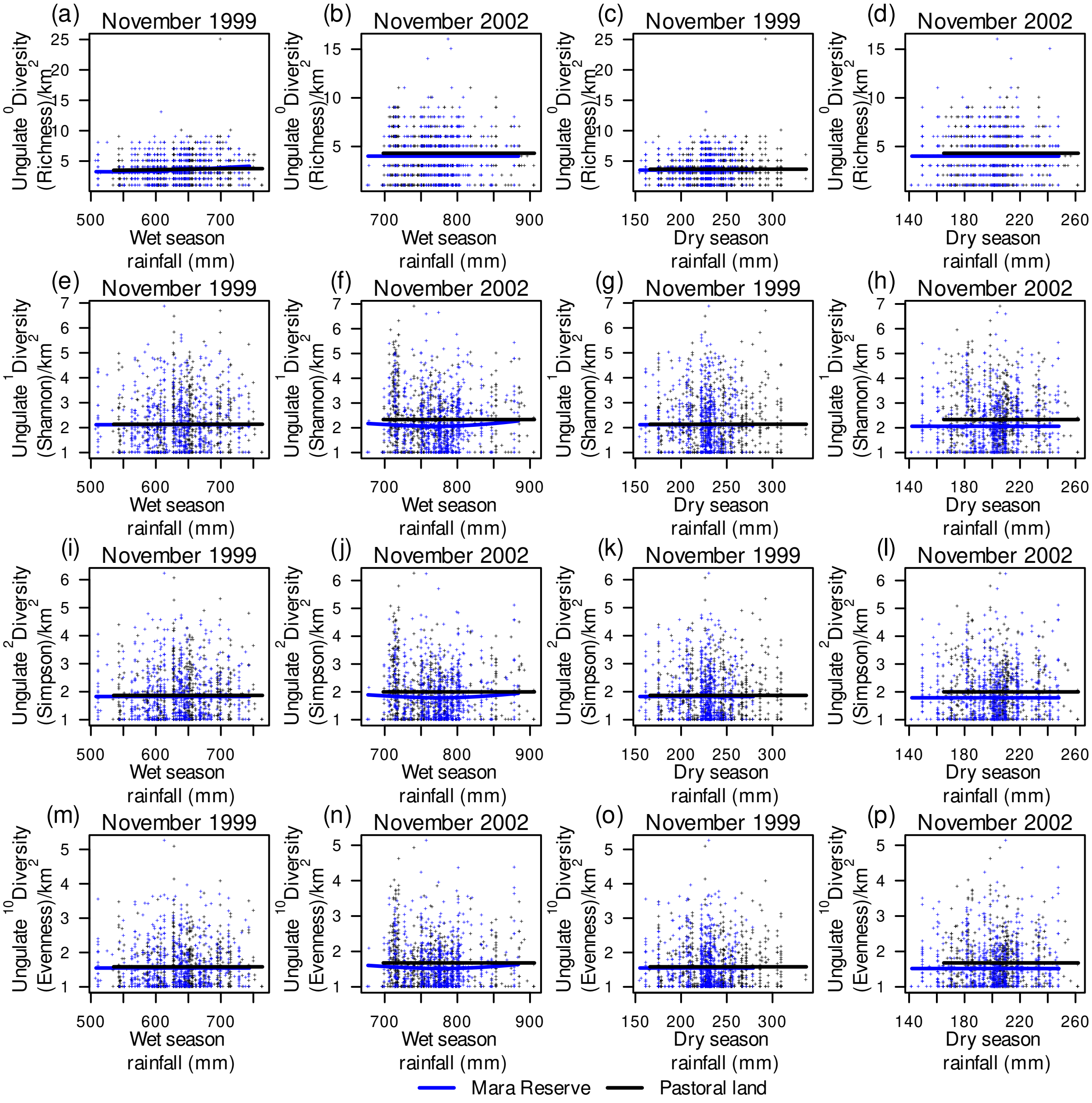

Figure S25: Ungulate diversity based on orders 0 (richness in a-d), 1 (Shannon in e-h), 2 (Simpson in i-l) and 10 (species evenness in m-p) in relation to the wet season rainfall (a,b,e,f,i,j,m,n) and the dry season rainfall (c,d,g,h,k,l,o,p) in the Maasai Mara National Reserve (blue lines for predictions and blue pluses for observations) and adjacent pastoral lands (black lines for predictions and black pluses for observations) in Kenya in November of the 1999 drought year (a,c,e,g,i,k,m,o) and November of the 2002 normal rainfall year (b,d,f,h,j,l,n,p).

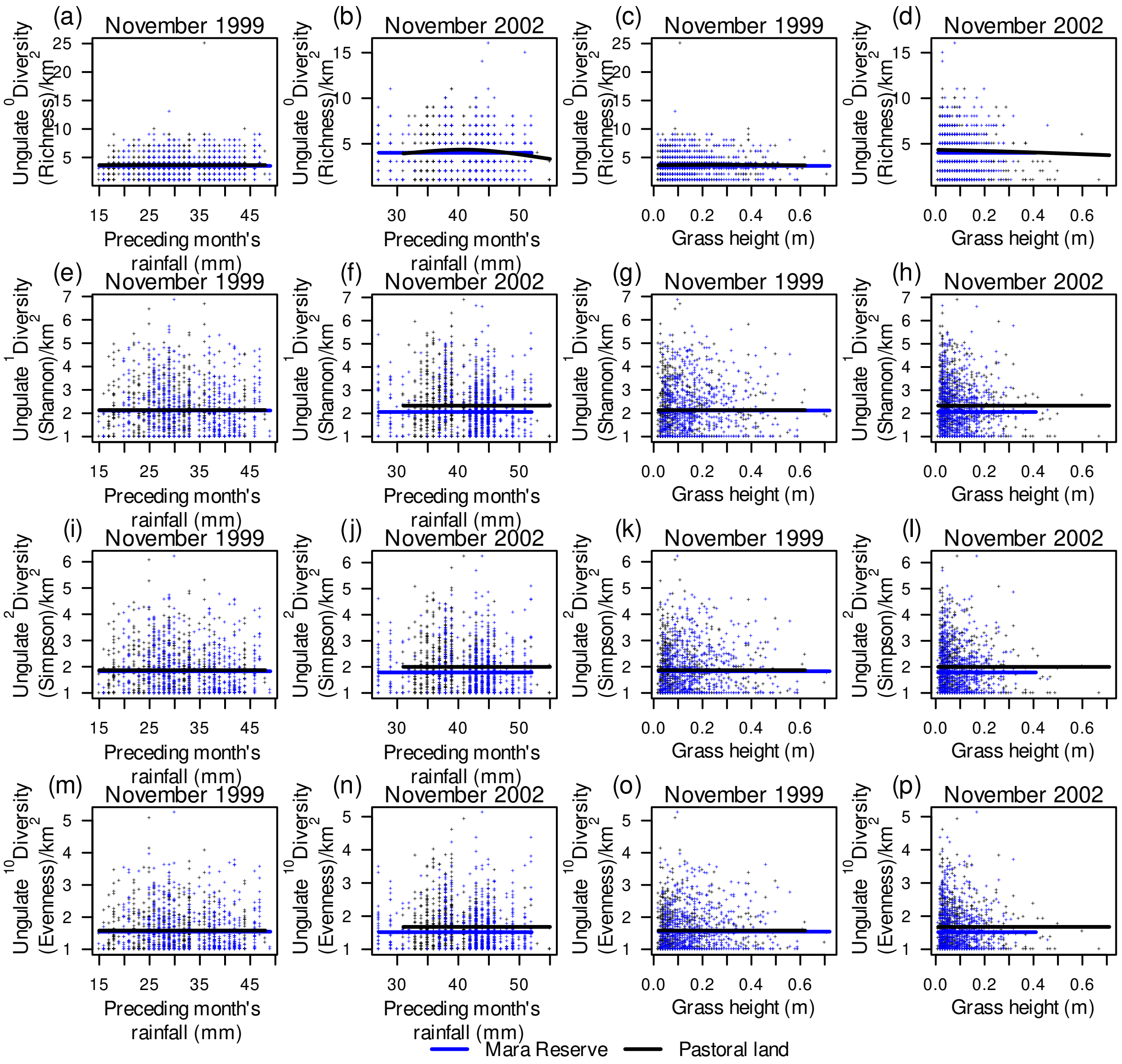

Figure S26: Ungulate diversity based on orders 0 (richness in a-d), 1 (Shannon in e-h), 2 (Simpson in i-l) and 10 (species evenness in m-p) in relation to the preceding month's rainfall (a,b,e,f,i,j,m,n) and grass height (c,d,g,h,k,l,o,p) in the Maasai Mara National Reserve (blue lines for predictions and blue pluses for observations) and adjacent pastoral lands (black lines for predictions and black pluses for observations) in Kenya in November of the 1999 drought year (a,c,e,g,i,k,m,o) and November of the 2002 normal rainfall year (b,d,f,h,j,l,n,p).
