# Supplementary material for: Balancing Savanna Ungulate Diversity and Biomass: Optimal Human Use, Landscape Features, and Vegetation Types Under Varying Rainfall and Land Use
Source: Ecol Evol. 2026 Apr 20;16(4):e73501. doi: 10.1002/ece3.73501 (PMC13095871; doi:10.1002/ece3.73501)
Supplement: Supplementary file 2 — Appendix S2: ece373501‐sup‐0002‐AppendixS2.pdf. [file ECE3-16-e73501-s001.pdf]

Appendix S2

Balancing Savanna Ungulate Diversity and Biomass: Optimal Human use, Landscape Features and Vegetation Types Under Varying Rainfall and Land Use

Ecology and Evolution

Gundula S. Bartzke, Joseph O. Ogutu, Hans-Peter Piepho, Claire Bedelian, Michael E. Rainy, Russel L. Kruska, Jeffrey S. Worden, Kamau Kimani, Michael J. McCartney, Leah Ng'ang'a, Jeniffer Kinoti, Evanson C. Njuguna, Cathleen J. Wilson, Richard Lamprey, N. Thompson Hobbs, Robin S. Reid

Contents

Section S1: Extended Results **2**
Section S1.1: Grass 2
Section S1.2: Slope and Elevation 2
Section S1.3: Shrubs and Trees 3

S1: Extended Results

S1.1: Grass

In both census years, raw species richness peaked at 12 to 15 units of grass color score in the Mara Reserve (Figures 1e,f; Appendix S1: Figures S1i,j). Bias-adjusted species richness peaked within the same range of grass color scores in the reserve in 1999, while both raw and bias-adjusted species richness peaked at these values on pastoral lands in 2002 (Figures 1e,f; Appendix S1: Figures S1j, S11a,b). Bias-adjusted species richness increased by 45% and diversity measures accounting for evenness increased by between 8% and 10% as grass color scores increased from 5 to 18 in the Mara Reserve in 2002 (Figure 1f; Appendix S1: Figures S11b,f,j,n). Shannon diversity increased by 9% with increasing grass color scores from 3 to 18 on pastoral lands in 2002 (Figure 1f; Appendix S1: Figure S11f).

S1.2: Slope and Elevation

Ungulate diversity, except for species evenness in 2002, peaked on low slopes between 2° and 3° in the Mara Reserve in 1999 and on pastoral lands in 2002 (Appendix S1: Figures S15a,b, S16a,b, S17a,b,e,f,i,j,m). In 1999, raw species richness decreased by 85% as slope increased from 0° to 13° on pastoral lands and ungulate biomass decreased by 19% as slope increased from 0° to 10° in the Mara Reserve (Appendix S1: Figures S15a, S16a,c). Ungulate diversity in both census years and ungulate biomass in 1999 peaked between 1,628 masl and 1,701 masl on pastoral lands (Appendix S1: Figures S15c,d, f, S16e-g, S17c,d,g,h,k,l,o,p). Ungulate biomass decreased by 70% as elevation increased from 1,540 masl to 1,849 masl on pastoral lands in 2002 (Appendix S1: Figures S15d, S16h).

S1.3: Shrubs and Trees

Ungulate biomass increased 5.2-fold as shrub cover increased to 39% in the Mara Reserve and 2.5-fold as tree cover increased to 50% on pastoral lands in 1999 (Figures 3c,e; Appendix S1: Figures S12g,k). Raw ungulate species richness decreased by 73% with increasing shrub cover up to 95% and bias-adjusted species richness decreased by 33% with increasing shrub cover up to 67% in the Mara Reserve in 2002 (Figure 3d; Appendix S1: Figures S12f, S18b). On pastoral lands, raw species richness decreased by 17% as shrub cover increased up to 67% in 2002 (Figure 3d; Appendix S1: Figure S12f). Ungulate diversity of higher orders than bias-adjusted species richness increased by between 8% and 14% with increasing shrub cover up to 67% in the Mara Reserve in 2002, and ungulate diversity of all orders increased by between 4% and 17% with increasing shrub cover up to 47% in the reserve in 1999 (Figures 3c,d; Appendix S1: Figures S18a,e,f,i,j,m,n).

Ungulate diversity decreased by between 6% and 26% as tree height increased from 2.8 m to 8.6 m on pastoral lands in 2002 (Appendix S1: Figures S13f, S14j, S20b,f,j,n). Ungulate biomass decreased by 65% with increasing tree height up to 6.4 m and then increased 2.9-fold as tree height increased from 6.4 m to 13.0 m in the Mara Reserve in 2002 (Appendix S1: Figures S13f, S14l).

Bias-adjusted ungulate diversity increased by between 4% and 12% as shrub color score increased from 2 to 18 on pastoral lands in 2002 (Appendix S1: Figures S21b, S19c,g,k,o). Ungulate diversity, excluding species evenness, in 1999 and raw species richness in 2002 peaked at 10 to 14 tree color units in the Mara Reserve (Appendix S1: Figures S21c,d, S22i,j, S20c,g,k). Species richness and Shannon diversity increased by between 5% and 36% as tree color increased from a score of 4 to 18 on pastoral lands in 1999 (Appendix

S1: Figures S21c, S22i, S20c,g).
